# XABOOM: An X-ray Absorption Benchmark of Organic Molecules Based on Carbon, Nitrogen, and Oxygen 1s → π* Transitions

**DOI:** 10.1021/acs.jctc.0c01082

**Published:** 2021-02-05

**Authors:** Thomas Fransson, Iulia E. Brumboiu, Marta L. Vidal, Patrick Norman, Sonia Coriani, Andreas Dreuw

**Affiliations:** † Interdisciplinary Center for Scientific Computing, Ruprecht-Karls University, Im Neuenheimer Feld 205, 69120 Heidelberg, Germany; ‡ Fysikum, Stockholm University, Albanova, 10691 Stockholm, Sweden; § Department of Theoretical Chemistry and Biology, KTH Royal Institute of Technology, 10691 Stockholm, Sweden; | Department of Chemistry, Korea Advanced Institute of Science and Technology, 34141 Daejeon, Korea; ⊥ DTU Chemistry, Technical University of Denmark, Kemitorvet Bldg 207, DK-2800 Kongens Lyngby, Denmark; # Department of Chemistry, NTNU-Norwegian University of Science and Technology, N-7991 Trondheim, Norway

## Abstract

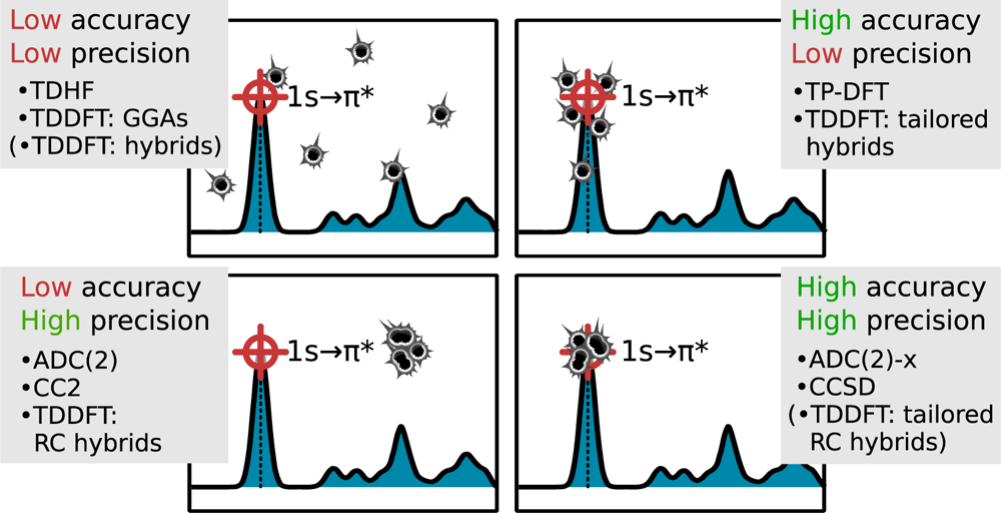

The performance of several standard and popular approaches for calculating X-ray absorption spectra at the carbon, nitrogen, and oxygen K-edges of 40 primarily organic molecules up to the size of guanine has been evaluated, focusing on the low-energy and intense 1s → π* transitions. Using results obtained with CVS-ADC(2)-x and fc-CVS-EOM-CCSD as benchmark references, we investigate the performance of CC2, ADC(2), ADC(3/2), and commonly adopted density functional theory (DFT)-based approaches. Here, focus is on *precision* rather than on *accuracy* of transition energies and intensities—in other words, we target relative energies and intensities and the spread thereof, rather than absolute values. The use of exchange–correlation functionals tailored for time-dependent DFT calculations of core excitations leads to error spreads similar to those seen for more standard functionals, despite yielding superior absolute energies. Long-range corrected functionals are shown to perform particularly well compared to our reference data, showing error spreads in energy and intensity of 0.2–0.3 eV and ∼10%, respectively, as compared to 0.3–0.6 eV and ∼20% for a typical pure hybrid. In comparing intensities, state mixing can complicate matters, and techniques to avoid this issue are discussed. Furthermore, the influence of basis sets in high-level *ab initio* calculations is investigated, showing that reasonably accurate results are obtained with the use of 6-311++G**. We name this benchmark suite as XABOOM (X-ray absorption benchmark of organic molecules) and provide molecular structures and ground-state self-consistent field energies and spectroscopic data. We believe that it provides a good assessment of electronic structure theory methods for calculating X-ray absorption spectra and will become useful for future developments in this field.

## Introduction

In recent times, the field of X-ray spectroscopy has progressed rapidly as a result of the development and construction of modern synchrotrons and X-ray free-electron lasers, enabling the investigation of light–matter interactions at unprecedented time resolution and radiation intensity.^1,2^ These installations facilitate the study of exotic molecular properties and provide a sensitive experimental tool to questions such as (i) probing chemical reactions in real time, as exemplified by the tracking of the photocatalytic cycle in photosynthesis,^2−6^ (ii) considering the structure of molecular samples, such as the local structure of liquid water,^7−9^ (iii) identifying the oxidation state of transition metals in organometallic complexes, with examples including the Fe/Mo atoms in nitrogenase,^10−12^ (iv) investigating nonlinear properties, such as stimulated emission and two-photon absorption,^13−17^ and more. Using pump–probe protocols, time-resolved spectroscopies can study a multitude of dynamical processes, but this potential is yet to be fully explored due to the significant theoretical and experimental difficulties of performing such studies, with, for instance, experimental facilities only being made available during the last decade. The modeling of transient X-ray spectroscopy is a relatively new field, with one of the first systematic studies of transient X-ray absorption and emission spectroscopy from as late as 2015.^18^ Nonetheless, the field has experienced rapid growth, encompassing applications ranging from photodissociation^19,20^ and ring-opening reactions^21,22^ to excited state dynamics,^23,24^ intersystem crossings,^25,26^ and many more.^2,3,27,28^

In order to interpret and understand these advanced measurements, an interplay between the experiment and computational chemistry is required. In time-resolved measurements, however, a comparison between the experiment and theory is not necessarily straightforward. For the purpose of benchmarking the underlying theoretical methods, it thus makes good sense to limit oneself to steady-state properties. In this study, we therefore address the modeling of X-ray absorption spectroscopy (XAS), in which the excitation of core electrons to bound or continuum states is probed. Transitions to bound states generally provide information on the unoccupied states, while transitions to the continuum probe the atomic structure of the sample. These subfields of XAS are referred to as the near-edge X-ray absorption fine structure (NEXAFS) or X-ray absorption near-edge spectroscopy and extended X-ray absorption fine structure (EXAFS). In this study, the modeling of the NEXAFS is considered, that is, the transition of core electrons to bound states—see refs^29−31^ for general discussions of this spectroscopy from both a theoretical and experimental perspective. Compared to the field of theoretical spectroscopy in the UV/vis region, for which numerous extensive benchmark studies are available (see, e.g., ref (32)), systematic comparisons on the performance of methods for calculating X-ray absorption spectra are still rather sparse. Of note is the work of Besley and Asmuruf,^33^ who investigated the performance of time-dependent density functional theory (TDDFT) for core properties and the construction of functionals with reasonable absolute energies, and numerous smaller studies for different methods.^34−44^

In order to accurately model core excitation processes, the inclusion of electron relaxation effects is vital. This requires a theoretical method capable of capturing two physical effects: a reduced screening of the probed nuclei following the removal of a core electron, leading to a strong net attraction of the electron density toward the core and a smaller repulsive polarization effect in the valence region due to the interaction with the excited electron. These counteracting effects need to be properly accounted for in a theoretical framework, either by explicitly optimizing the excited state or by introducing at least doubly excited configurations. Furthermore, relativistic effects are important for spectroscopies targeting core electrons, on account of the strong potential experienced by these electrons, and they scale strongly with the atomic number. For electrons occupying s-orbitals, these effects are scalar in nature and easily accounted for. By comparison, for electrons occupying orbitals with *l* > 0, there will be strong spin–orbit coupling effects that are nontrivial in general and necessary to rigorously include in the Hamiltonian.^45^ For transition metal complexes, multiplet effects must also be considered.^46^ Furthermore, in the case of heavy elements, the electric dipole approximation also becomes progressively worse, with quadrupole-allowed transitions becoming more intense as *Z* increases.^47,48^ However, it is well justified to neglect both spin–orbit couplings and quadrupole-induced transitions at the K-edges of light elements, as investigated here.

An abundance of methodologies for modeling X-ray absorption spectra have been developed, including semi-empirical, density-based, and wave function-based methods.^31,33,34,36−39,43,45,49−68^ Here, the focus is on first-principles methods, and semi-empirical approaches will thus be left out of the discussion. Among the first-principles methods, researchers in the field of theoretical spectroscopy commonly apply DFT. However, while DFT offers many advantages—particularly in terms of computational costs—its predictability is precarious, especially when considering systems and processes for which suitable exchange–correlation functionals have not yet been identified. These issues are enhanced for TDDFT when addressing core excitations and nonlinear properties, owing to issues relating to self-interactions and lack of two-electron excitations.^31,33,69−72^ Furthermore, DFT was originally formulated to capture both the correct densities and energies, but contemporary functionals often focus almost solely on energies, thus achieving a smaller energy error at the cost of larger density errors.^73^ Therefore, the development and application of *ab initio* wave function methods for computational spectroscopy continue to be vital, especially for novel applications. Nonetheless, TDDFT has been successfully used to model XAS and other X-ray spectroscopies,^33,74−79^ and tailoring exchange–correlation functionals for core properties is an active field of research.^33,80−89^ Alternatively, an approach based on Slater’s transition state method has been developed, called transition potential DFT (TP-DFT), which explicitly considers fractionally occupied core and (potentially) valence orbitals, thus accounting for the largest contributions to electron relaxation.^65^ This method has been successfully applied to numerous spectrum calculations,^50,65,90−92^ although the issue of exact fractional occupation continues to be debated.

Moving to wave function-based methods, single- and multireference methods have both been used, and electron relaxation can be accounted for through electron correlation by the use of (at least) doubly excited configurations.^34−36,51,53,54,56,68,93−98^ Available schemes include single-reference methods such as coupled cluster (CC) theory, the algebraic diagrammatic construction (ADC) approach, density cumulant theory, and multireference methods such as RASSCF, RASPT2, and MR-CC. Regardless of the underlying electronic structure method used, an issue for any method based on molecular response theory is the embedding of core-excited states in the continuum of valence-excited states, which makes a straightforward application of an iterative diagonalization scheme such as the Davidson algorithm^99^ unfeasible for all but the smallest of systems. One solution is to neglect the coupling between the valence- and core-excited states, thus effectively including only states with at least one core electron excited. This is based on the very weak couplings due to large energetic and spatial separation between core and valence states and it is referred to as the core–valence separation (CVS) approximation.^94^ The scheme has been successfully implemented in several electronic structure methods, with the detailed algorithms varying somewhat.^31,54,58,74,96,100,101^ The error introduced by this approximation has been shown to be small and independent of the compound.^54,102,103^ It can be accounted for by the use of perturbation theory^54,103^ or relaxation of the CVS eigenstates^102^ or circumvented entirely by the use of damped response theory,^104−108^ real-time propagation schemes,^56,76^ or adapted Lanczos algorithms.^34,109^

In the present work, the performance of several first-principles methods commonly used for modeling X-ray spectroscopies is evaluated. We focus on the carbon, nitrogen, and oxygen 1s → π* transitions of small- and medium-sized organic molecules, as these absorption bands are low-lying, intense, and distinct, allowing for an (almost) unambiguous comparison. Our emphasis is placed on *precision* rather than *accuracy*, as it is difficult and often not of main concern to pinpoint exact transition energies in experiments but rather study relative energies, energy shifts, and intensities imposed by the local structure and dynamics. Furthermore, the energy scale under investigation is in the region of several hundreds of electronvolts, and to reach an accuracy in absolute energy similar to what is reached in the valence region is neither to be expected nor vital. The employment of overall energy shifts of theoretical spectra typically does not limit the applicability of the methods but rather corrects for *systematic* absolute errors that may vary significantly for the different elements due to differences in relaxation and self-interactions. The key here is that a method shows systematicity in errors (i.e., precision). We coin this benchmark XABOOM (X-ray absorption benchmark of organic molecules) and provide underlying spectroscopic data and molecular geometries and self-consistent field (SCF) energies in the Supporting Information. We quite obviously encourage the use of this benchmark for a critical assessment of methods used for calculating conventional NEXAFS spectra. However, more broadly, it is also relevant for more advanced studies such as time-resolved pump–probe experiments, as long as the core-excited states are of a single-electron transition character. The dynamic molecular structure must also remain in regions where the ground state is of single-reference character; otherwise, multireference state approaches are needed. We refer to recent studies for illustration of this broader applicability and limitations of propagator approaches under such circumstances.^59,110^

The outline of this study is as follows: First, we briefly discuss the most popular approaches for simulating X-ray absorption spectra of organic molecules, with an emphasis on TDDFT, TP-DFT, ADC, and CC. We then present the molecules selected for XABOOM and discuss our selection of spectral bands and choice of reference values. Following a specification of computational details, we present our results together with a detailed discussion before providing our conclusions. The discussion includes the topics of selecting appropriate reference values, choosing basis sets, and identifying distinct and separate spectral features.

### Theoretical X-ray Absorption Spectroscopy

In this section, we will briefly describe the most popular approaches to calculate X-ray absorption spectra of organic compounds at the carbon, nitrogen, and oxygen K-edges. Besides those chosen for the present benchmark study, there are numerous other methods that are used in the communities of computational chemistry and material science.^31^ Most notably, our selection refers exclusively to single-reference methods and they therefore suffer from the associated and well-known limitations. Inarguably, multireference approaches—such as the multiconfiguration self-consistent field method,^111^ with its complete^112^ and restricted active space (RASSCF) variants,^113^ including the respective perturbation theory CASPT2 and RASPT2 extensions^114,115^—represent indispensable tools for strong (or static) correlation. The XABOOM molecules, however, do not belong to such cases and the use of multireference methods with the accompanying selection of active spaces and separate-state optimizations of a large number of core-excited states is generally not required. For these systems, it is a better alternative to use unbiased polarization propagator or linear response theory approaches, often offering a systematic route toward higher precision and accuracy.

The price to be paid in polarization propagator-based approaches is the more indirect treatment of electronic relaxation and polarization in the valence shell. It can therefore be worthwhile to consider approaches that relax the electron density in the core-excited state to a varying degree. One such alternative is the static-exchange approximation (STEX)^49,67^ that employs a common set of relaxed orbitals for the configuration interaction singles (CIS) formation of the entire set of excited states at a given edge. STEX has historically had an important role in evaluating experimental spectra,^31,50,116,117^ but it has fallen out of use due to issues relating to lack of electron correlation and spectrum compression^31,35,50,118^—while STEX accounts for the dominant relaxation effect arising from the creation of a core hole, the lack of the weaker polarization effects yields term values that are too small and thus compresses the spectra in a manner that affects separate core-hole sites differently. Other choices are the several variants of TP-DFT techniques that are described in a bit more detail below and which have been assessed in the present benchmark.

### Transition Potential DFT

The most straightforward way to describe core excitations is to make use of the fact that the dipole operator is a single-electron operator and to rewrite the linear response function in terms of the spin orbitals of the variationally relaxed ground state.^119^ This is applicable to single-Slater determinant methods, such as Hartree–Fock (HF) or Kohn–Sham (KS) DFT, and entails the computation of transition matrix elements between the 1s-orbital and unoccupied molecular orbitals (MOs) in the ground state. This is a drastic approximation that completely neglects orbital relaxation and results in a poor agreement with experimental data, when it comes to both peak positions and intensities.^92,120^ Relaxation effects can instead be included using Slater’s transition state method^121,122^ or by setting the occupation of the core level of interest to 0.5 and relaxing the electronic structure in the presence of this half core hole (HCH). Used in combination with DFT, this method is known as TP-DFT.^65,123^ By additionally introducing a shift such that the eigenvalue of the core level is equal to the calculated ionization energy (IE) (ΔKS correction), TP-DFT provides XAS spectra that compare well to experiment in many cases,^50,65,90,123−125^ albeit with some occasional difficulties in sufficiently capturing relaxation effects.^92^ This motivated the use of a full core-hole on the core-excited atom^92^ or alternatively a full core hole in combination with an electron placed in the lowest unoccupied MO (excited-state core hole).^91^ Owing to the low computational cost of TP-DFT, X-ray absorption spectra of rather large molecules can be calculated with reasonable accuracy in comparison to experiment.^92,126,127^ However, TP-DFT is essentially a ground-state single-particle approach, where orbital relaxation is not included rigorously but via the adjustable core-hole occupation parameter.

### Linear-Response TDDFT

The next step in going beyond a ground-state theory for XAS is to write the equation of motion for the linear response of the system of electrons to the applied electromagnetic field. This is achieved in time-dependent HF (TDHF) and TDDFT by introducing the random-phase approximation (RPA) operator^117^
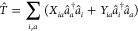
1where indices *i* and *a* refer to occupied and unoccupied orbitals, respectively, *â*^†^ and *â* are the creation and annihilation operators, respectively, and **X** and **Y** are the excitation and de-excitation vectors obtained from the RPA equation, respectively^76,117,128,129^
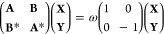
2with
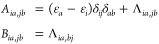
Here, the term Λ collects the anti-symmetrized two-electron integrals (e.g., Λ_*ia*,*jb*_ = (*ia*∥*jb*)).^129^ Besides the response of the Coulomb potential to the perturbation, Λ contains the responses of the HF exchange potential (in TDHF) and the approximate exchange–correlation (xc) potential (in TDDFT).^129^

It should be noted that the de-excitation vector is generally understood as introducing a portion of ground-state electron correlation in the RPA formulation.^117,129^ By neglecting this term, that is, removing **Y** from eq 1 and setting matrix **B** in eq 2 to zero, the Tamm–Dancoff approximation (TDA) is introduced.^130^ In TDHF, this approximation is equivalent to CIS.^129^ The RPA or TDA matrix can be diagonalized within the CVS approximation, generating the core excitation energies, excitation vectors, and related properties. Because only single excited determinants are included, relaxation effects are unaccounted for in TDHF and CIS. This leads to a significant overestimation of transition energies, as the final state is not sufficiently relaxed and thus too high in energy. The description of XAS can be improved by using a HF core-ionized ground state as a reference for the CIS Hamiltonian, which is the basic idea in the STEX approach.^49,67,117^

In TDDFT, the correlation effects giving rise to the relaxation effects can, at least in principle, be accounted for. Due to the approximate nature of the xc-functional, however, TDDFT suffers from self-interaction errors (SIE)^31,33,69,70,131^ that are exacerbated in the case of core excitations and which have spurred the design of a plethora of tailored xc-functionals.^31,33,131^ These include global functionals where the amount of exact exchange is optimized with respect to core excitations, for example, B^0.58^LYP,^88^ and functionals with state-specific exact-exchange corrections, for example, CVR-B3LYP.^87^ Also, range-separated hybrid functionals have been employed in XAS with notable success in reducing both the absolute and relative error with respect to experimental data seen for SRC1 and SRC2,^88^ BmLBLY,^85^ LCgau-BOP,^86^ and CAM(100%)-B3LYP.^107^ Optimally tuned range-separated functionals, where the range separation parameter and amount of exact exchange are tuned for a particular system to fulfill a physically motivated condition, such as the ionization potential (IP) theorem, have also shown promise in the description of core excitations.^89^ Another related strategy has been to obtain optimal parameters for one molecule by enforcing the IP theorem with respect to experimental IP values for several orbitals and then use these parameters for all other systems in a universal type of xc-functional, as exemplified by the range-separated CAM-QTP00^80^ and global QTP17^82^ functionals. These different schemes for improving the description of core excitations typically achieve significantly improved absolute energies, but the performance in terms of element-dependent relative energies is less investigated. Note that the lack of appropriate relaxation and the self-interaction effects partially cancel, such that a pure generalized gradient approximation (GGA) functional underestimates carbon K-edge transition energies by ∼18 eV, which can be compared to the typical TDHF overestimation of ∼9 eV. Achieving appropriate absolute energies thus largely becomes a matter of tuning the amount of exact exchange, such that these counteracting effects cancel.

### Algebraic Diagrammatic Construction for the Polarization Propagator

Turning to correlated *ab initio* approaches to describe excited states, one alternative is provided by the ADC scheme, in which a perturbation expansion of the matrix representation of the polarization propagator is constructed and the excitation energies and vectors are obtained by matrix diagonalization.^103,132^ An intuitive way to construct the ADC matrix and the associated working equations is provided by the intermediate state representation approach,^133−135^ introducing the Hamiltonian (*Ĥ*) matrix shifted by the ground-state energy (*E*_0_) in the basis of a set of intermediate excited states
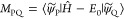
3

The intermediate states are essentially obtained by applying the excitation operator *Ĉ*_P_ = {*â*_*a*_^†^*â*_*i*_;*â*_*a*_^†^*â*_*b*_^†^*â*_*i*_*â*_*j*_;...} to the Møller–Plesset reference state. Excitation energies (Ω_*n*_) and excitation vectors (**X**_*n*_) are obtained from the eigenvalue equation
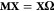
4

The ADC hierarchy is defined by truncating the perturbation expansion at a desired order. Since this truncation is also related to the excitation classes used to obtain the ADC matrix elements, the size of the ADC matrix depends on the truncation order. ADC(1) is obtained by truncating the series at the first order and including only single excitations—this makes it equivalent to CIS as far as energies are concerned. ADC(2) goes up to the second order in perturbation theory and includes both single and double excitations so that relaxation effects are largely accounted for.^36^ A further extension to ADC(2) is the ad hoc description of the doubles block up to the first order of perturbation theory in the extended ADC(2) or ADC(2)-x approach.^103,132^ This improves the description of double excitations and, therefore, also of orbital relaxation in core excitation calculations.^36^ The rigorous description of the doubles block up to the first order is achieved at the level of ADC(3/2), where the singles block is described up to the third order and the couplings block up to the second order.^132^ Within the CVS approximation, ADC schemes up to the third order have been implemented to describe core excitations of closed-^36,51^ and open-shell systems.^52^ As such, ADC has been successful at describing X-ray absorption spectra for a large number of systems, ranging from small molecules, such as diatomics,^95,103^ to medium-sized and large molecules, such as nucleobases,^36,136,137^ porphin, and PTCDA.^51^

### Coupled Cluster Methods for Excited States

An alternative hierarchy of propagator methods can be defined based on CC theory. Here, the starting point is the CC reference state, |CC⟩ = e^*T̂*^|0⟩, typically constructed from the HF state |0⟩ and the cluster operator *T̂* = *T̂*_1_ + *T̂*_2_ + .... Truncation of the cluster operator at a given level defines a hierarchy of CC methods: CC singles (CCS), CC singles and doubles (CCSD), and so on.^138^ An intermediate CC2 level of theory is further obtained from CCSD by including the double excitations only up to the first non-zero term in perturbation theory.^139^ Comparing corresponding levels of ADC and CC theory, the computational scaling of the latter is slightly higher since the reference-state amplitudes are determined iteratively. The formal scaling of CC2 and ADC(2) is the same, with ADC(2) being correct to one order higher for response properties. CCSD is correct to one order higher in perturbation theory than ADC(2) for ground-state energies and double excitations and to the same order for single excitations and response properties and it scales as *n*^6^, same as ADC(2)-x.^132^

Starting from the CC reference state, excitation energies and excitation vectors can be obtained either via linear-response (LR-CC)^140−142^ or equation-of-motion (EOM-CC) formulations.^143,144^ These approaches are closely related and both require the diagonalization of a non-Hermitian Jacobian matrix **A** with elements *A*_μν_
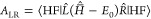
5
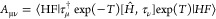
6where *L̂* and *R̂* are excitation operators typically truncated at the same level as *T̂* and  is the similarity-transformed Hamiltonian.^138^ Since the Jacobian is asymmetric, the eigenvalue problem is solved for both left (**L**) and right (**R**) eigenvectors^142,145^
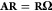
7
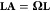
8

The core excitations embedded in the eigenvalue equations mentioned above can be targeted and reached employing the CVS approximation.^54,58^ With this, CC has been successfully applied to describe XAS spectra for small- and medium-sized molecules, showing high accuracy in comparison to experimental data (see, e.g., refs (34, 35, 40, 43, 53, 109, 146)

### Molecular Systems and Selected States

**Figure 1:**
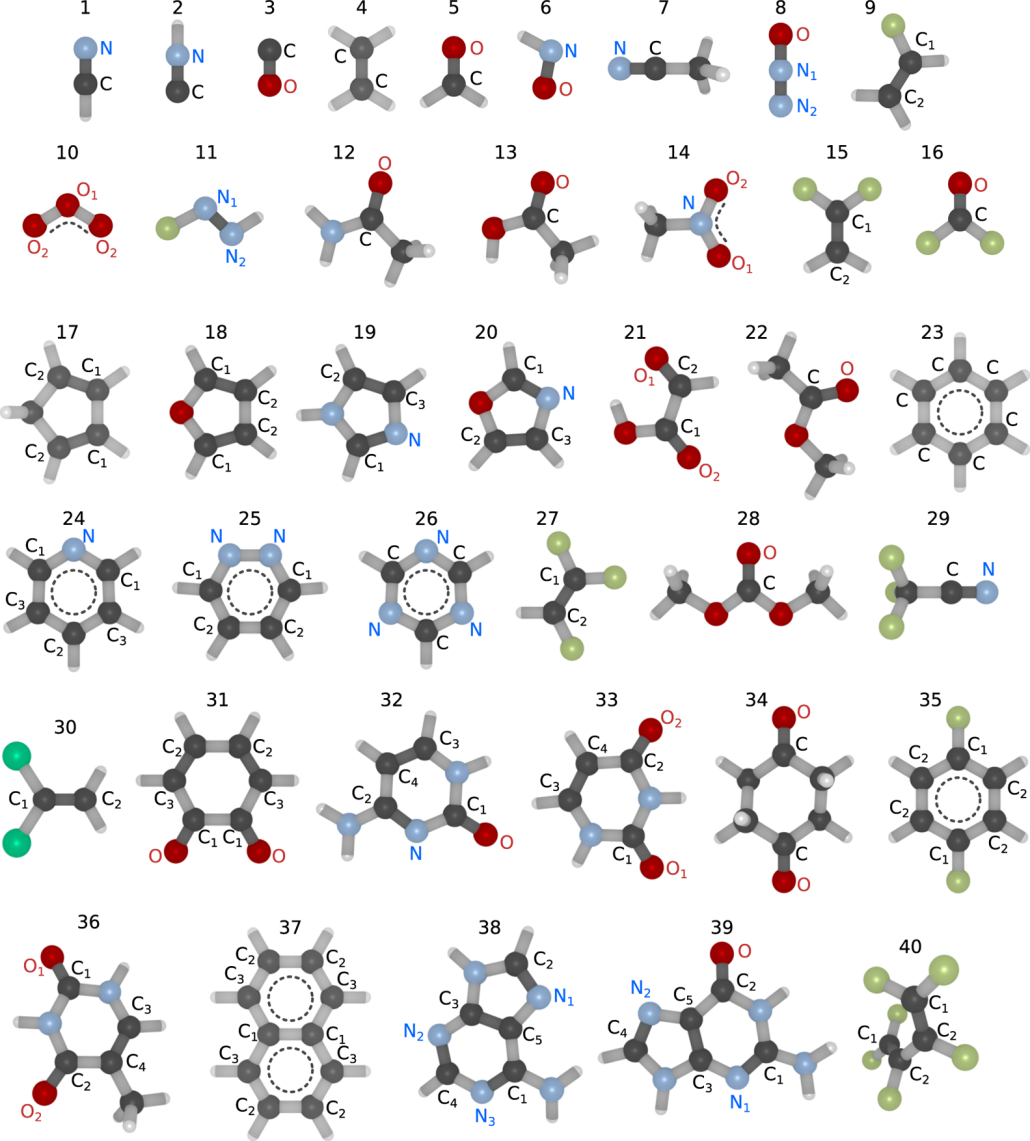
Molecules included in the XABOOM benchmark set, ordered according to molecular mass. Atoms participating in double bonds are labeled, with chemically inequivalent atoms of the same species identified by indices. Single bonds are colored in light gray, double/triple bonds are colored in dark gray, and delocalized double bonds are marked with dotted lines.

For the XABOOM benchmark set, we have selected 40 primarily organic compounds including unsaturated aliphatic hydrocarbons, heterocycles, aromatic hydrocarbons, carbonyl compounds, nucleobases, and more, as illustrated in Figure 1. This selection is inspired by the renowned benchmark set of Thiel and co-workers^147,148^ and is meant to be representative of the chemical space most interesting for spectroscopic studies of organic compounds.

The investigated bands comprise 1s → π* transitions at the carbon, nitrogen, and oxygen K-edges, focusing on local transitions from atoms involved in bonds of order higher than one. As an example, only transitions from the C=O carbon and oxygen in acetic acid (compound 13) are considered and thus labeled in Figure 1. This selection serves multiple purposes: (i) the π*-resonances give rise to strong and relatively narrow spectral features, making them suitable for experimental–theoretical comparisons; (ii) XAS is increasingly used for solutions, where, for example, Rydberg features are quenched by the environment and thus not suitable for analysis; (iii) in surface science, the π*-resonances are highly polarization-dependent and thus provide a probe for orientation of adsorbed molecules and structured systems; (iv) these features are relatively easy to identify in our calculations, albeit with a risk of state mixing, which will be discussed below. Similar focus on π*-resonances has previously been conducted for, among others, carbonyls,^149^ substituted benzenes,^50^ and ethenes.^35^ For systems where Rydberg states are of interest, the adopted TDDFT approaches are expected to vary in accuracy,^33,38,86−88^ and the *ab initio* wave function methods are likely to have more extensive basis set requirements.

With a focus on local transitions, we have opted for a basis set that adds diffuse functions only to atoms directly involved in double or triple bonds, thus improving the description of transitions into the π* space. We have selected cc-pVTZ as a main basis set for non-hydrogen atoms, augmented to aug-cc-pVTZ when participating in higher order bonds. Hydrogens are described with cc-pVDZ. This basis set selection is labeled as aT/T/D, and a comparison to results using a basis set of quadruple-ζ quality is performed for the most accurate methods included here.

Due to difficulties in obtaining appropriate experimental reference values (as discussed in detail below) and inability of obtaining full configuration interaction estimates for the systems investigated, we instead chose to use fc-CVS-EOM-CCSD and CVS-ADC(2)-x as our references, which have been demonstrated to yield results in very good agreement with experiments for small- and medium-sized systems.^36,43,51−53,55−59^ Using both methods and paying attention to cases where they show noticeable discrepancies, we achieve theoretical estimates of sufficient quality for this benchmark study. Should more accurate methods capable of considering both transition energies and intensities be made computationally affordable for the systems considered here, we encourage a future critical assessment of our selection. Recent developments at the levels of CC3 and multilevel CC could provide such a path forward.^150,151^ For brevity, the CVS/fc-CVS prefixes will henceforth be dropped, but it is to be understood that all ADC results have been obtained using the CVS scheme presented in ref (51) and the EOM-CCSD values have been obtained using the fc-CVS approach established in ref (58).

## Computational Details

The molecular structures have been optimized at the frozen-core MP2^152^/cc-pVTZ^153^ level of theory, using the Gaussian program.^154^ The results are available as xyz-files in the Supporting Information. Property calculations have been performed employing versions of the Dunning family of basis sets,^153^ including augmentation with diffuse^155^ and core-polarizing^156^ functions, with additional basis set investigations using the Pople 6-311++G**^157^ basis set. All property calculations have been performed using a nonrelativistic framework.

The ADC calculations using a common CVS space have been performed in the adcman^158^ module of Q-Chem^159^ 5.1, employing the tensor library libtensor.^160^ The EOM-CCSD calculations have been performed using the ccman2 module of Q-Chem 5.2 employing the same libtensor library. Note that in the fc-CVS-EOM-CCSD approach adopted here, the core orbitals relevant in the CVS-EOM step are kept frozen during the optimization of the ground-state wave function parameters (amplitudes and multipliers). For the ADC calculations using CVS spaces tailored to specific atoms, the adcc^161^ package was used, utilizing pyscf^162^ for obtaining SCF references.

The TDDFT results were obtained using Q-Chem 5.1, using global and range-separated hybrid functionals including PBE,^163^ B3LYP,^164^ BHandHLYP,^165^ B^0.58^LYP,^88^ rCAM-B3LYP,^166^ CAM-QTP00,^167^ SRC2-R1,^88^ and a modified version of CAM-B3LYP with 100% exact exchange in the asymptotic limit (hence referred to as CAM100%).^107,168^ The DFT parameterizations used are included in the Supporting Information for clarity (Table S2).

The CC2 calculations were performed with the Dalton package,^169,170^ adopting a CVS variant where the core and valence excitations are decoupled in the target space^54^ only, that is, the ground-state wave function contains excited-state determinants involving both core and valence orbitals. This choice was made to keep a close resemblance with the CVS algorithm used here for the ADC(2) method. CC2 calculations could be performed indifferently with either common or tailored CVS spaces.

The TP-DFT calculations were performed using the PBE functional^163^ in StoBe 2014^171^ and the BHandHLYP functional^165^ in PSIXAS.^172^ For the latter, we use the designation HCH (BHH) in figures and tables for the sake of brevity. For each probed atom in the molecule, an individual XAS spectrum was calculated by relaxing the electronic structure in the presence of a HCH localized on that particular atom. In StoBe, orbital rotations of the core orbitals on centers other than the active one were restricted in order to localize the HCH. PSIXAS instead makes use of the maximum overlap method (MOM),^173^ which yields issues relating to spatially delocalized HCHs for systems with delocalized MOs. As such, effective core potentials of the Stuttgart/Cologne group (ECP2MWB)^174^ were used for all atoms of the same species, save the core-excited one. We adopted the Dunning basis sets for consistency, and tests using PBE in StoBe have shown that this combination yields minimal difference when compared to results using orbital rotation restrictions. For each core-excited atom, the IE was calculated by taking the energy difference between the ground state and a core-ionized state localized on the active site (ΔKS). Each atom-specific spectrum was then shifted such that the eigenvalue of the 1s-orbital in the HCH approximation matched the calculated IE.

## Results and Discussion

This section is organized as follows: First, we consider the basis set effects of the most computationally demanding approaches included here, that is, ADC(2)-x and EOM-CCSD, with a focus on error spreads. We then deliberate on our choice of reference values, followed by a discussion on state mixing and how this can be minimized. The results of the full XABOOM benchmark set are then presented. We have chosen to focus on a graphical presentation of the results, but tabulated values and raw data can be found in the Supporting Information.

The statistics used for our analysis are the mean error (ME), standard deviation (SD), and maximum (absolute) deviation (MD). They are given as
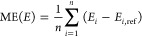
9
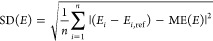
10
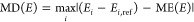
11
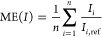
12
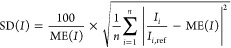
13
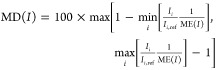
14

For energies, this simply corresponds to the relative error and distribution thereof. For intensities, the different methods can have noticeably different oscillator strengths but similar scaling when considering different systems and states. As such, we focus the intensity discussion on ratios, with error spreads and maximum deviations expressed as percentages from the baseline ratio. Individual ratios of, for example, 0.81 and 0.99 thus both represent a deviation of 10% from a baseline intensity ratio of 0.90.

### Basis Set Effects

**Figure 2:**
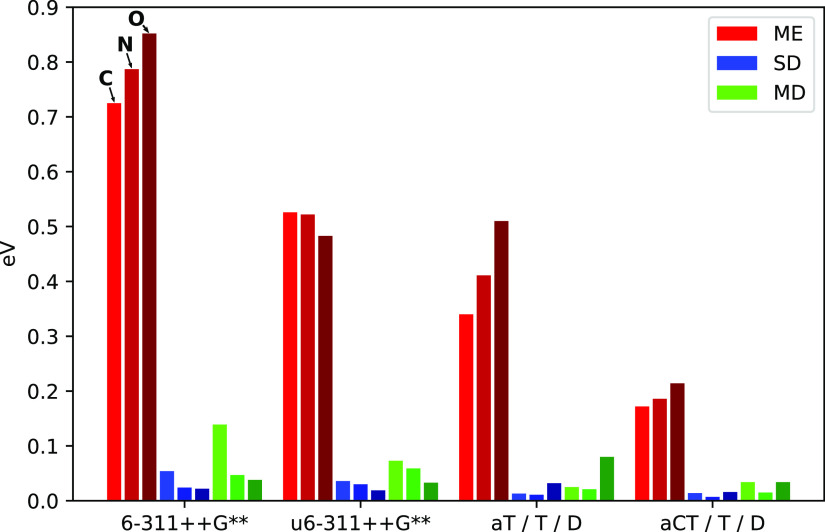
Basis set effects at the level of ADC(2)-x, as obtained by comparison to aug-cc-pCVQZ/cc-pVQZ results. Reporting ME, SD, and MD, as defined in eqs 9–11. The statistics have been obtained from 14 transitions for carbon, 10 transitions for nitrogen, and 10 transitions for oxygen.

The basis set requirements of ADC(2)-x and EOM-CCSD have been considered for a subgroup of the full XABOOM benchmark set, with results for ADC(2)-x illustrated in Figure 2. Tabulated values and ADC(2)-x and EOM-CCSD results for a smaller set of molecules are included in the Supporting Information. These two methods are the most advanced and computationally challenging approaches included here, and as such, the basis set requirements of the remaining methods are expected to be smaller. This is particularly the case for TDHF and TDDFT,^175^ where the effects have been shown to amount to a change in the ME and SD of ≤0.05 eV for the addition of core-polarizing functions to an aug-cc-pVTZ basis set^37^ or when going from aug-cc-pCVTZ to aug-cc-pCVQZ.^176^

Using aug-cc-pCVQZ/cc-pVQZ with the diffuse functions added to atoms participating in double or triple bonds as a reference, we have investigated the performance of the adopted aT/T/D basis set and of three others. First, we augmented the main selection with core-polarizing functions on the probed atoms, yielding aCT/T/D. Second, we investigated two versions of the 6-311++G** basis set—one using the standard form and one where the 1s CGTOs for carbon, nitrogen, and oxygen are decontracted (designated as u6-311++G**). The latter has been noted to yield improved agreement with experiment over the standard 6-311++G** basis set, which is already in good agreement with experiment,^36^ at a relatively small increase in computational cost.^44^

As expected, we obtain decreasing absolute and relative errors when adding flexibility in the core region or when moving to correlation-consistent Dunning basis sets. With aCT/T/D, the ME amounts to 0.17–0.21 eV, with a spread of 0.01–0.02 eV, which increases to 0.34–0.51 and 0.01–0.03 eV, respectively, when core-polarizing functions are removed. Removing the core-polarizing functions furthermore introduces an element dependence in the MEs, as core relaxation effects scale with the atomic number. For LR-CCSD, the difference between core-polarized triple- and quadruple-ζ levels has been reported as 0.11–0.13 eV for the π*-resonances of formaldehyde, thus slightly lower than that observed for our set of molecules.^43^ With aug-cc-pVXZ basis sets, the difference between TZ and QZ has been reported as 0.3 eV using ADC(3/2), with an additional correction of 0.1 eV when moving to 5Z.^36^ In general, amending standard basis sets with core-polarizing functions has proven to be a relatively cheap way of increasing the accuracy for core properties, as these properties require flexibility in the core region. This extra flexibility can be achieved by use of specially tailored functions, using functions from the next element or decontracting the tightest contracted basis functions.^35,44,58,175,176^ Using the 6-311++G** basis set, the decontraction of the 1s CGTO results in lowering of MEs by 0.2–0.4 eV and largely removing element dependencies. The maximum error for carbon also decreases noticeably, while remaining maximum errors and SDs are within 0.03 eV of 6-311++G** results. Overall, the error spread of the basis sets considered here is below 0.06 eV, and the maximum deviations never exceed 0.14 eV. Adding core-polarizing functions to our main basis set combination decreases error spreads with at most 0.02 or 0.05 eV in maximum error. In terms of relative intensity, the largest deviation observed is only 0.98 ± 0.01. Results for EOM-CCSD are consistent with the ones of ADC(2)-x, with larger MEs (10–50%) and slightly increased error spreads.

For the basis set used in the remainder of this study, the largest discrepancies are 0.51 ± 0.03 and 0.73 ± 0.03 eV for ADC(2)-x and EOM-CCSD, respectively, as noted for the oxygen edge. We conclude that all of these basis sets are reasonable choices for probing 1s → π* transitions, with the introduced error primarily amounting to a potentially element-dependent shift in absolute energy. Note that if other transitions are probed—particularly Rydberg or mixed Rydberg states—the basis sets used here may no longer be sufficient.

### Choice of Reference Values

Ideally, we would benchmark theoretical methods either against very high-level theoretical estimates or against experimental data. The former would, however, significantly affect the size of systems and basis sets under consideration, and comparison to experiments comes with its own difficulties. Additionally, high-level theoretical results are in some cases available for transition energies but not for intensities,^93^ and here, we seek to evaluate the performance for both properties. As such, the results presented here are compared to reference values obtained using EOM-CCSD and ADC(2)-x, two methods which have been shown to yield good agreement with experiments on top of having a solid theoretical foundation.^24,36,43,51−59^ Note that these methods yield accurate relative features, albeit with element-dependent absolute errors—as an example, the discrepancies with respect to experiments for CO/HCN/CO amount to approximately −0.1/–0.6/–1.1 eV for ADC(2)-x and 0.3/0.7/1.2 eV for EOM-CCSD, with relativistic effects accounted for.

Comparisons to experimental XAS data are complicated due to the following issues: (i) Straightforward application of theory yields vertical transition energies, which are often not a good representation of the experimental features.^32^ (ii) Spectral features will overlap in regions where the density of states is high, making it difficult to isolate individual transitions. (iii) Systems can exhibit significant tautomerism, which scrambles the spectra even more.^137^ (iv) Experiments are often done in an environment, which can become very cumbersome to consider accurately in computations. (v) Even for gas-phase spectra, vibrational effects can make the comparison less than straightforward. (vi) Relativistic effects can be highly influential, especially for transitions from *l* > 0 where an appropriate treatment of spin–orbit coupling is needed to get correct branching ratios.^45,78^ (vii) Available experimental spectra occasionally differ noticeably from each other, as a result of varying setups, calibrations, and other factors. For example, the carbon π*-resonance of acetone has been reported as 286.80 and 286.44 eV, with a smaller discrepancy of 0.08 eV for the oxygen K-edge.^177,178^ For the carbon edge of formaldehyde, a difference of 0.11 eV can be found,^177,179^ and disparities of 0.3 eV between the oxygen energies of CO and O_2_ or 0.5 eV when looking purely on O_2_ have been noted.^178^ We stress that trends and relative features are often more important than absolute values, with spectra for a compound or for a set of compounds investigated under the same experimental conditions, yielding highly reliable results.

### Isolating Individual Excited States

Isolating the individual 1s → π* transitions is for most systems a simple task, but for systems with close-lying states, there can be significant mixing, which complicates matters. In Figure S1, we illustrate the ADC(2)-x and EOM-CCSD carbon spectrum of acetamide (system 12). The peak maximum has two intense contributions at the EOM-CCSD level, while that obtained with ADC(2)-x has only one. This is because the C=O π*-resonance in EOM-CCSD strongly mixes with a transition from the methyl carbon, while for ADC(2)-x, the features are more separated in energy. A direct comparison between these reference methods would thus yield a large intensity discrepancy. These intensity sharing effects have been observed for several systems in the XABOOM benchmark set, in particular for heterocyclic compounds for which the density of states can be high and near degeneracies are present. We note that this excited-state mixing can potentially affect spectrum assignments, as it may give weak transitions unrealistically high intensities and thus lead to assignments which significantly overestimate the influence of these transitions. Controls using a protocol such as the following can thus be important for evaluating X-ray absorption spectra.

In order to avoid issues with state mixing, we have used a simple approach: for cases where mixing may occur, additional calculations are performed, considering each atom individually. In TP-DFT, this is already done, so no mixings are present in these results. For TDDFT, this is done by restricting the allowed channels (CVS space) to one core MO at a time, and for ADC and CC2, it is also possible to use such a tailored CVS space.^161^ For EOM-CCSD, we instead fed the algorithm with guess vectors, considering transitions from each unique MO in turn. Using tailored CVS spaces is technically not in line with the CVS approximation, for which the coupling between valence- and core-excited states is neglected for spatial and energetic reasons. We have ensured that the approach does not introduce new artifacts by extensive testing, an example of which can be found in Figure S1. For EOM-CCSD, there is a small shift in transition energies of the mixing states (<0.02 eV), but the general features are barely affected. The intensity sharing is removed, and the π*-resonance is seen to have most of the total intensity. For ADC(2)-x, there is hardly any difference between the case where both carbon atoms are considered simultaneously or individually. For both ADC(2)-x and EOM-CCSD, the difference in integrated intensity in a region of ±1.5 eV from the π*-resonance is less than 0.5%, and the effect is thus considered to be well within the acceptable range. A tailored CVS space or manual guess vector is thus used for systems with state mixing involving a π*-resonance. This approach has an additional advantage for some of the larger systems: converging all states up to the highest-lying π*-resonance can occasionally be quite challenging, and directly targeting the core MOs in question circumvents this issue. Finally, the tailored CVS spaces should not be and have not been used for delocalized core MOs.

## Benchmark Results and Discussion

**Figure 3:**
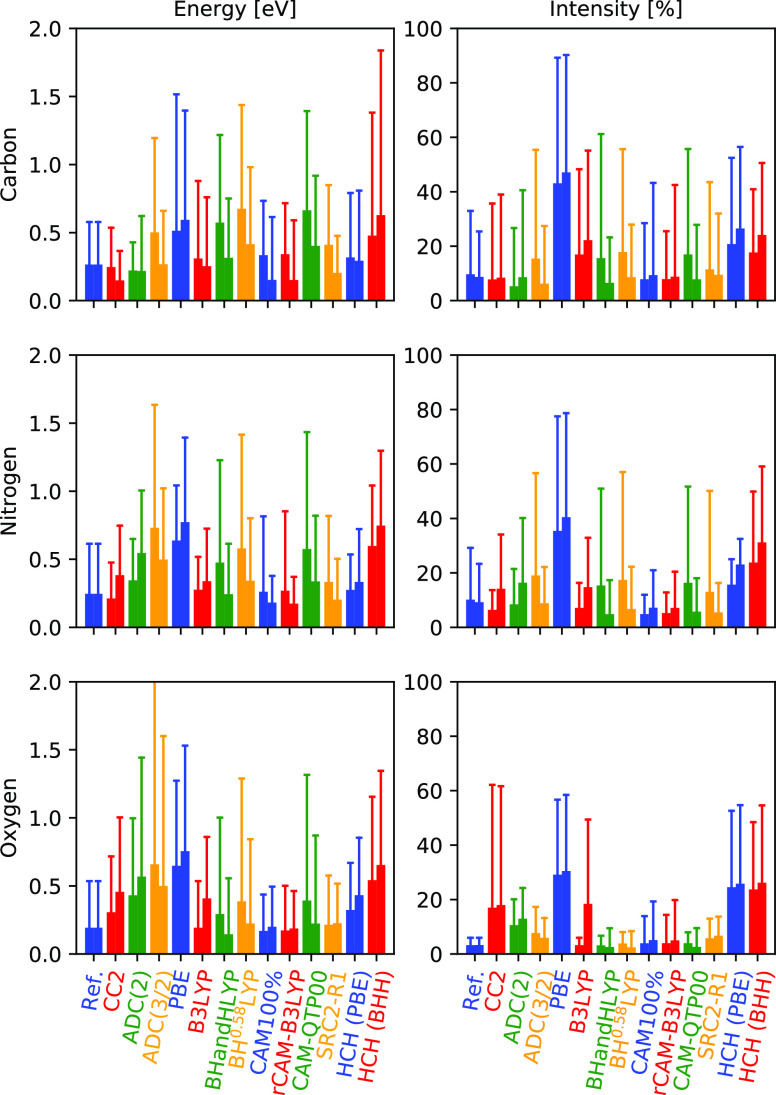
Performance of 13 methods for calculating 1s → π* transition energies and intensities, as compared to ADC(2)-x (left bars) and EOM-CCSD (right bars) reference values. Left-most results (labeled Ref.) compare the reference values to each other. Showing error spreads (bars) and maximum deviations (lines). See eqs 9–14 for definitions of statistics.

**Figure 4:**
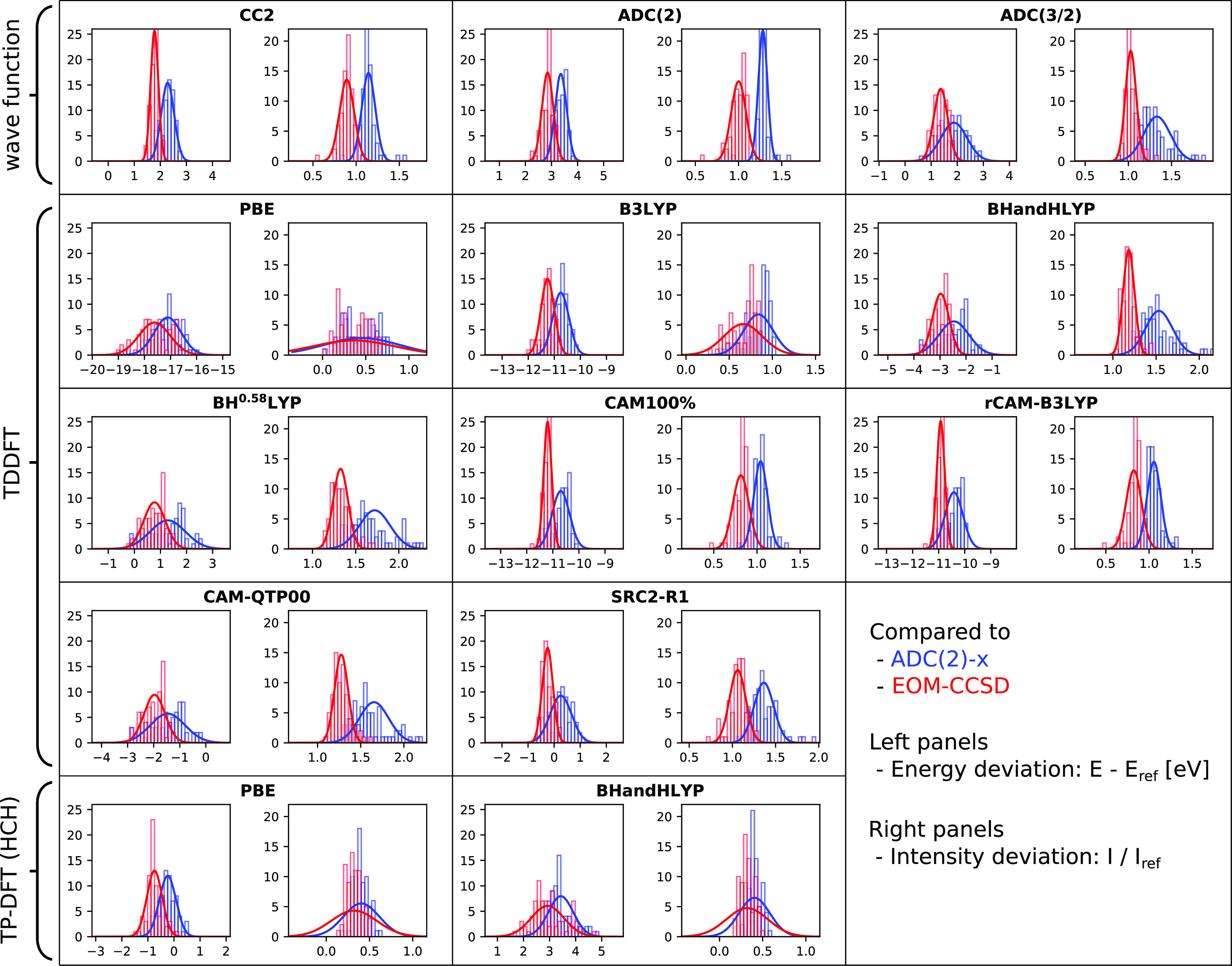
Relative performance of 13 methods for calculating carbon 1s → π*, as compared to ADC(2)-x (blue) and EOM-CCSD (red) reference values. Showing histograms and Gaussian distributions constructed from SDs (eqs 10 and 13), with intensity discrepancies here expressed as ratios in order to provide information on absolute intensity differences. Left panels show discrepancies in energies and right panels show discrepancies in intensities.

**Figure 5:**
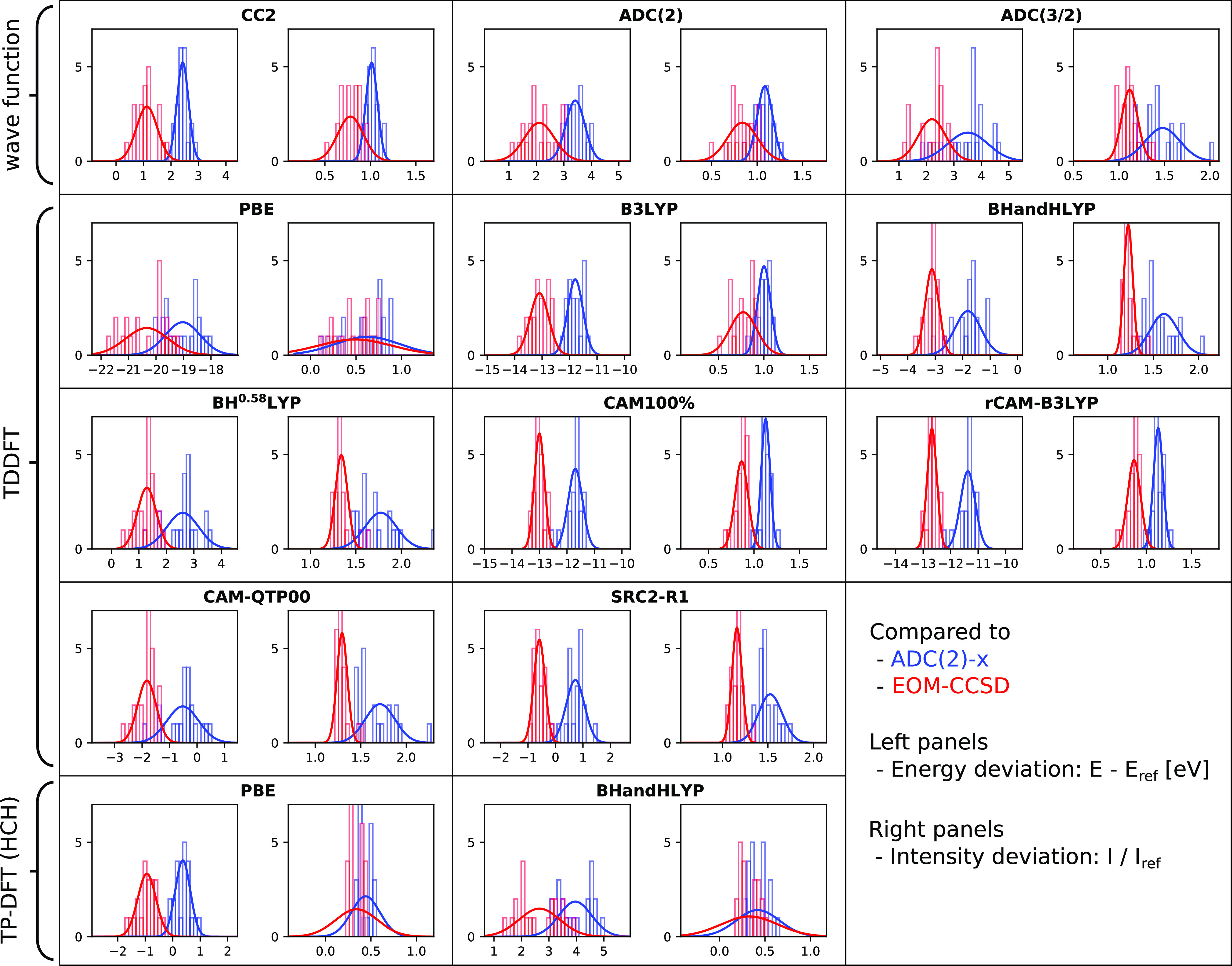
Relative performance of 13 methods for calculating nitrogen 1s → π*, as compared to ADC(2)-x (blue) and EOM-CCSD (red) reference values. See Figure 4 for details.

**Figure 6:**
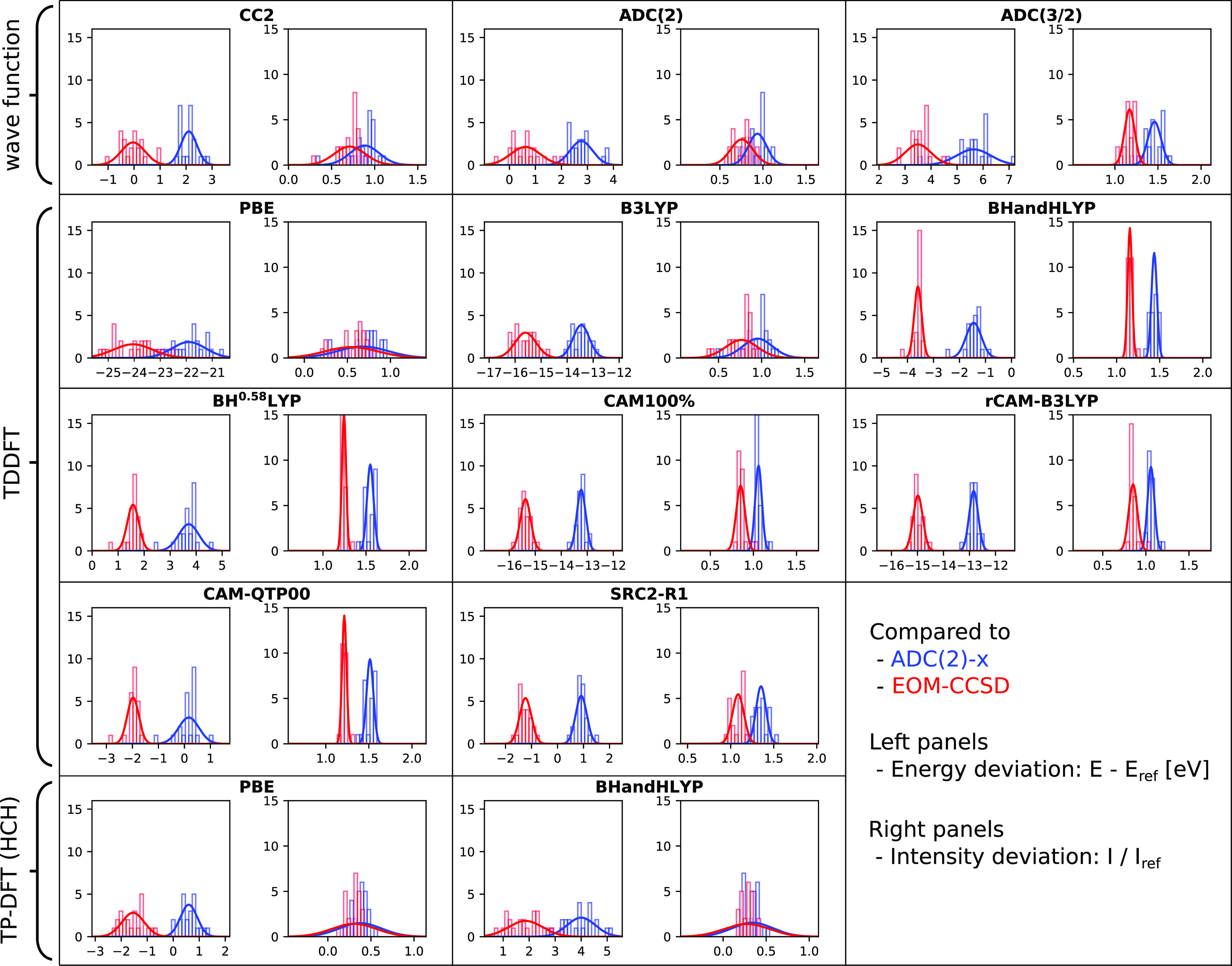
Relative performance of 13 methods for calculating oxygen 1s → π*, as compared to ADC(2)-x (blue) and EOM-CCSD (red) reference values. See Figure 4 for details.

We here focus on a visual presentation of the results, showing error spreads and maximum deviations in Figure 3 and histograms in Figures 4–6. The weighted average error spreads are also reported in Table 1, but these averages do not contain information on any element dependencies. The total number of states is 72, 21, and 23 for carbon, nitrogen, and oxygen, respectively. The results are tabulated in the Supporting Information, also including MEs.

**Table 1:** Error Spread of Excitation Energies and Intensities, as Compared to ADC(2)-x and EOM-CCSD^a^

	ADC(2)-x		EOM-CCSD	
	*E*	*I*	*E*	*I*
ADC(1)	1.15	24	0.91	15
ADC(2)	0.29	7	0.35	11
ADC(2)-x			0.25	8
ADC(3/2)	0.57	15	0.36	7
CC2	0.25	9	0.25	11
CCSD	0.25	8		
PBE	0.56	39	0.66	43
B3LYP	0.30	15	0.30	20
BHandHLYP	0.50	13	0.27	5
B^0.58^LYP	0.60	15	0.36	7
CAM100%	0.29	7	0.17	8
rCAM-B3LYP	0.29	7	0.16	8
CAM-QTP00	0.59	14	0.36	6
SRC2-R1	0.36	11	0.21	8
HCH (PBE)	0.31	21	0.33	26
HCH (BHH)	0.51	20	0.65	26

a Showing average spreads over all elements, weighted with the number of respective transitions (e.g., factor 72/116 for the carbon error spreads). Energy spread expressed in eV and intensity spread in %.

### Comparison of Reference Methods

In general, the results in Figure 3 show the error spreads to be quite consistent between using ADC(2)-x or EOM-CCSD as a reference. The maximum deviations vary a bit more, often showing a larger difference to the ADC(2)-x reference set. The error spreads and maximum deviations in transition energies between ADC(2)-x and EOM-CCSD amount to 0.19–0.27 and 0.54–0.61 eV, respectively. For intensities, the discrepancies amount to 3–10% and 6–33%, using ADC(2)-x as a reference. Considering only molecules with up to five heavy (non-hydrogen) atoms, as included in the Supporting Information, shows marginal differences in SDs, and comparing the quadruple-ζ results obtained for our basis set investigation also shows similar error spreads. The present values of approximately 0.25 eV and 9% error spread in energy and intensity, respectively, are thus considered to be close to the converged values and can be regarded as to be the maximum “resolution” of the present benchmark set—further analysis has to be carried out with this in mind. Scatter plots illustrating the results of EOM-CCSD and ADC(2)-x are provided in the Supporting Information, where results of particular interest (larger discrepancies or extremal energies/intensities) are labeled. The largest energy deviation is found for the nitrogen edge of system 14 (nitromethane), and the largest intensity deviations were found for C_2_ of system 32, that is, the carbon bonded to an amine group in cytosine. Matthews compared EOM-CCSD and EOM-CC3 excitation energies to those obtained by EOM-CCSDT, yielding element-specific error spreads of 0.24–0.29 eV for EOM-CCSD and 0.03–0.13 eV for EOM-CC3.^93^ These values are in line with the present error spreads.

In terms of absolute values, the discrepancies between ADC(2)-x and EOM-CCSD increase from 0.51 to 2.14 eV when moving from carbon to oxygen, with ADC(2)-x results being lower in energies. For intensities, EOM-CCSD oscillator strengths are 25–31% more intense, with no clear trend following the atomic number. For the energies, ADC(2)-x underestimates experimental values and EOM-CCSD overestimates them—as lack of relaxation leads to too high transition energies, this suggests that ADC(2)-x overestimates the influence of reduced screening of the probed nuclei and EOM-CCSD underestimates it. The overestimation of the former comes from the somewhat ad hoc manner in which the ADC(2)-x doubles block is extended to the first order in perturbation theory, which results in more noticeable contributions from doubly excited configurations, as seen by a larger *T*^2^ value for ADC(2)-x compared to the other ADC methods or CC. Nonetheless, the present results suggest that both approaches are more or less on equal footing for the calculation of X-ray absorption spectra, and by always comparing to both reference values, we believe that our analysis of the more approximate methods is strengthened.

### Wave Function-Based Methods

We have included results from the ADC hierarchy of methods (ADC(1), ADC(2), and ADC(3/2)) and the intermediate CC2 method. The ADC(1) results are not included in the illustrations, but the mean spread is found in Table 1, and tabulated results are found in the Supporting Information. Note that ADC(1) is identical to CIS and CCS for energies, and all these methods give very similar results as the more expensive RPA (or TDHF). The MEs obtained by ADC(1), which amount to ∼9–17 eV, are thus representative for all these methods and, for the purposes of this study, represent results lacking electron relaxation effects. Previous studies have reported relaxation effects differing by about 12 eV for the bare atom and up to almost 15 eV for larger aromatic systems.^136^ The intensities are furthermore exaggerated by up to 156%, with somewhat lower values for oxygen. More importantly, the error spreads and maximum errors are large, amounting to 0.82–1.19 eV and 5–30% for spreads and 3.17 eV and 142% for maximum deviations. Correspondence to EOM-CCSD is in all cases better than to ADC(2)-x, likely a result of ADC(2)-x overestimating relaxation effects and thus moving further from HF. These large errors and error spreads are expected and represent the lower limit in terms of accuracy and precision, as obtained with an uncorrelated and unrelaxed approach.

Moving to ADC(2) and CC2, the resulting spreads and maximum deviations are relatively similar—this is not unexpected, as the two methods are very related by construction and comparable conclusions have been drawn also for valence properties.^180^ The error spreads in energies are larger for ADC(2), in particular compared to EOM-CCSD. For the intensities, the error spreads of carbon and nitrogen are similar, but they deviate quite significantly for oxygen. In general, deviations for the oxygen K-edge are larger for the wave function-based methods, particularly for energies, and dimethyl sulfoxide (DMSO) in particular creates large deviations for ADC(2) and CC2. This system is not included in the XABOOM benchmark set but is considered in the Supporting Information. ADC(2) and CC2 here yield maximum deviations of close to 1.5 eV compared to ADC(2)-x or over 2 eV compared to EOM-CCSD. These discrepancies are not present for systems such as SO and SO_2_, and it thus appears as if this particular structural motif provides additional challenges for the more approximate ADC(2) and CC2 methods. Maximum deviations of TDDFT also increase but not substantially. Furthermore, the inclusion of nitroxyl, nitrous oxide, and ozone (systems 6, 8, and 10, respectively) increases maximum deviations, and an example without these systems is also considered in the Supporting Information. With this, particularly the deviations in energies decrease across the board, and these structural motifs are thus understood to impose challenges as well, albeit not as substantial as DMSO. Intensities for oxygen show a generally low spread, except for CC2, and we note that our reference methods yield very good comparisons—this could be related to the relatively local character of the oxygen π*-resonances. Finally, the remaining larger intensity deviation for oxygen using CC2 is for system 34 (cyclohexadione), and the spread and maximum deviations of ADC(2) and CC2 are thus again very similar when this system is taken out of the analysis. Care thus needs to be taken in particular for the oxygen K-edge when ADC(2), CC2, or related methods are used. Still, the error spreads and maximum deviations using these relatively cheap methods are encouraging; in particular for the carbon K-edge, the error spreads amount to 0.15–0.25 eV and 5–9%, similar to the ones between ADC(2)-x and EOM-CCSD.

Finally, for ADC(3/2), the discrepancies to reference values are increased in almost all cases, save for oxygen intensities. ME spreads are seen to amount to 0.36–0.57 eV or 7–15%, with deviations in all cases being smaller when compared to those in EOM-CCSD. This is somewhat surprising, seeing that ADC(2)-x is an extension of ADC(2) with the doubles block taken from ADC(3). The poor performance of ADC(3/2) for core excitations has been analyzed in ref (36) and was attributed to a broken error cancellation at this level of theory. As such, ADC(3/2) is not recommended for the calculation of X-ray absorption spectra, as it yields noticeably worse results than ADC(2)-x, at a rather substantial increase in computational costs.

### Time-Dependent DFT

For TDDFT, we have included results for eight different exchange–correlation functionals, chosen to be representative of the functionals generally applied for calculations of X-ray absorption spectra. There exists a wide range of additional functionals in use,^80−82,86−88^ but we believe the selection here to provide results also applicable to many of these others.

First, one pure GGA (PBE) is included, primarily to show the performance of this functional class and to contrast to the TP-DFT results. Large errors are found in all cases, with error spreads of 0.51–0.77 eV and 29–47% and maximum deviations reaching upward to 1.53 eV and 90%. Such GGA functionals are not recommended for any production calculations, but they can be compared and contrasted to CIS in order to see what happens when exact exchange is added. The CIS overestimation of transition energies of 9–17 eV becomes an underestimation of 17–24 eV, and intensities reach from about two times larger than reference values to only half. The energy discrepancy results from the self-interaction counteracting the lack of relaxation, and balancing these two effects will be the approach to improve TDDFT results.

Second, we have included three global hybrids, namely, B3LYP, BHandHLYP, and B^0.58^LYP. The latter has been constructed to achieve accurate absolute core excitation energies for second-row elements and has been given 58% global nonlocal exchange. Increasing the amount of nonlocal exchange increases transition energies and intensities, resulting in changes of up to 17 eV. Focusing instead on error spreads and maximum deviations, the picture becomes less clear. B3LYP exhibits error spreads of 0.25–0.41 eV and 7–22%, BHandHLYP exhibits error spreads of 0.14–0.57 eV and 3–16%, and B^0.58^LYP exhibits error spreads of 0.22–0.68 eV and 2–18%. The results of the latter two functionals are generally close to each other, as they have rather similar parameters. The superior absolute energies of the more tailored functional do not necessarily translate to good relative features, with BHandHLYP generally performing slightly better. The error spreads obtained are similar to those reported by a previous study, where shifted SDs of 1.84, 0.55, and 0.51 eV were obtained for TDHF, B3LYP, and BHandHLYP, respectively.^37^ The smaller SDs obtained here, in particular for ADC(1)/TDHF, are largely a result of the fact that only π*-resonances are considered.

Third, four range-separated functionals were included, with three (CAM100%, rCAM-B3LYP, and CAM-QTP) long-range and one (SRC2-R1) short-range corrected. CAM100% is a modified version of the standard CAM-B3LYP functional with 100% exact exchange in the asymptotic limit, optimized for improved relative features in modeling core excitations.^107^ The rCAM-B3LYP functional is a refitted version of CAM-B3LYP, made to minimize many-electron self-interactions primarily for calculations of thermochemistry.^166^ These two functionals have a low degree of exact exchange in the short-range limit and thus exhibit large absolute errors. Constructed to minimize errors for inner-shell ionization energies,^167^ the CAM-QTP00 functional has a large degree of short-range exact exchange, with resulting core excitation energies within 2 eV from reference values. The SRC2-R1 functional is constructed differently, with a high degree of short-range exact exchange, which decreases with distance,^88^ to minimize absolute errors for calculations of X-ray absorption spectra. Accordingly, it yields absolute results closest to reference data. Focusing on error spreads and maximum deviations, however, we see that CAM100% and rCAM-B3LYP generally outperform the more tailored CAM-QTP00 and SRC2-R1 functionals, with error spreads in the order of 0.15–0.34 eV and 2–9%, being closer to EOM-CCSD than to ADC(2)-x results. The spreads are, in fact, smaller when compared to EOM-CCSD results than ADC(2)-x is to EOM-CCSD, with the sole exception of oxygen intensities. By comparison, the CAM-QTP00 results are very close to those of B^0.58^LYP, with the parameterization for short-range exchange being relatively similar. For SRC2-R1, the error spreads are improved but generally not as good as for CAM100% or rCAM-B3LYP.

As such, we conclude that tailoring exchange–correlation functionals for absolute energies does not necessarily lead to improved relative performance. In fact, the two functionals with the lowest relative errors—CAM100% and rCAM-B3LYP—have some of the highest absolute errors, however with absolute intensities close to reference values. SRC2-R1 also performs quite well and with absolute energies in close agreement with reference data. If a global hybrid is to be used, BHandHLYP is seen to perform best of the three such functionals included here.

### Transition Potential DFT

Finally, TP-DFT in the HCH formulation with the PBE exchange–correlation functional yields absolute energies close to reference values, being within 0.6 eV from the ADC(2)-x results. This is due to the relaxation effects being largely included in TP-DFT, with a ΔKS correction that brings the binding energy of the core level to the same value as the total energy difference between the ground state and the core-ionized state. PBE performs quite well when it comes to total and ionization energies,^181^ and transition energies are thus brought close to the reference values. Relative errors are also significantly improved over TDDFT using the same functional, although in particular, the intensity spreads still have a bit to go to reach the level of the best-performing functionals using TDDFT. Error spreads for energies are close to those of B3LYP, but for intensities, the error spread generally remains larger than that of ADC(1).

By comparison, the absolute energy errors using the BHandHLYP functional are larger than those of PBE by around 3 eV. This is not completely unexpected since the exchange and correlation functional^182^ and the choice of core-hole model^92^ both affect the energy position of the peaks. More surprisingly, the BHandHLYP results show larger error spreads than the PBE ones and a significant downgrade when compared to the TDDFT results using the same functional. With the HCH model, the balance obtained using a GGA functional and 0.5 occupation of the core level is thus disrupted when mixing in exact exchange. The roots of the HCH model can be traced back to Slater’s transition-state method, which made use of a 0.5 fractional occupation on particular states to obtain an approximation of the excitation energies.^183,184^ This approach was developed as approximate KS-DFT eigenvalues do not directly correspond to ionization energies or electron affinities and the dependence of the total energy with respect to occupation is not piecewise linear.^183,185^ LDA and GGA exchange–correlation functionals generate a convex deviation from piecewise linearity, in contrast to the HF method which generates a concave deviation.^185^ Thus, if the right amount of exact exchange is mixed with the GGA exchange, piecewise linearity can be restored. For such a corrected functional, it has been shown that the excitation energies are more correctly modeled using a full core-hole (or cation) model,^185^ and decreasing occupations of core levels with increasing amounts of exact exchange are required to balance the description of core-hole relaxation. The problem is that the amount of exact exchange which restores piecewise linearity is expected to be system- (and even orbital-) dependent,^186^ so the balance between the core-hole model and amount of exact exchange would also show this dependence. This presumably explains the broader distribution (higher error spread) of BHandHLYP results as compared to PBE ones. As such, the path toward improving TP-DFT results is less clear than for TDDFT. A more in-depth analysis of the relation between the functional and core-hole model is warranted but beyond the scope of the present study.

In terms of absolute intensities, the HCH values are approximately a factor of two lower than the reference values or about the same size for PBE using TP-DFT and TDDFT, while for BHandHLYP, they drop by about a factor of three compared to the TDDFT results. The lower intensity is thus related to the TP-DFT approach rather than the adopted exchange–correlation functional. We also note that TP-DFT requires the optimization of a state with a partially filled core hole, which has to be localized. Two different approaches of localizing this hole have been used in the literature: diagonalization of the *R*^2^ matrix in MO basis with respect to the probed atom^171^ and use of the MOM.^173^ For systems with core MOs localized to individual atoms, these approaches yield the same result, but for systems with delocalized MOs, the latter approach introduces an erroneous absolute shift in energy. Since TP-DFT (similar to STEX) has been derived for a core hole localized at a particular atom, the correct use of the MOM approach for highly symmetric systems involves breaking the symmetry and forcing the core hole to localize at a specific site (e.g., using ECPs or by increasing the charge of the core-excited atom by an infinitesimal amount). With this, TP-DFT remains a reasonable choice for very large systems due to its low computational cost.

## Conclusions

The performance of several quantum chemical single-reference methods for calculating X-ray absorption spectra at the carbon, nitrogen, and oxygen K-edge of (primarily) organic molecules up to the size of guanine has been evaluated, focusing on the low-energy and intense 1s → π* transitions. Using CVS-ADC(2)-x and fc-CVS-EOM-CCSD as our best theoretical estimates, we investigated the reliability of CC2, the ADC hierarchy, TP-DFT, and TDDFT using a suite of different exchange–correlation functionals. We have chosen to focus on *precision* rather than on *accuracy* or, in other words, on emphasizing the relative as opposed to absolute energies and intensities. This is largely due to the significant difficulties in reliably and effectively modeling electron relaxation effects, which result in noticeable errors in absolute energies even for high-level methods such as CCSD. This focus on relative features is particularly important for TDDFT, where these important relaxation effects are not included and the SIE leads to potentially large element-dependent errors in absolute energies.

With reference data sets that agree within 0.25 eV in energy or 9% in intensity, we investigated the performance of these methods for a set of 40 molecules, encompassing 116 individual transitions. We conclude that CC2 and ADC(2) perform relatively well, in particular for carbon, but care needs to be taken when probing oxygen π*-resonances. ADC(3/2) is shown to perform poorly both in terms of absolute and relative features, and a deeper analysis of this can be found in ref (36). Moving to TDDFT, we note that good performance in terms of absolute energies does not translate into good performance in terms of relative energies and see that long-range corrected functionals perform better compared to reference data. Error spreads are here brought down from approximately 0.3–0.6 eV in energy and ∼20% in intensity to 0.2–0.3 eV and ∼10%, respectively. The best-performing functionals are identified as CAM100% and rCAM-B3LYP, two versions of the standard CAM-B3LYP functional with (at least) 100% exact exchange in the asymptotic limit. The HCH formulation of TP-DFT noticeably improves upon the PBE results, but for BHandHLYP, it severely worsens the results obtained with TDDFT and even leads to larger error spreads than for PBE. This relates to a cancellation of effects stemming from the adopted functional and core-hole model and points to TP-DFT having a less-clear path of improvement than TDDFT. A core-hole model with smaller (and potentially variable) core level occupation is expected to better this situation, but a more in-depth analysis into this matter is beyond the scope of this study.

For the comparison of various methods, intensity borrowing between close-lying states is sometimes needed to be removed. We have shown that this can reliably be done using a tailored CVS space or guess vectors corresponding to transitions from chemically unique atoms. When comparing to experiment, this intensity borrowing is not important when considering total (convoluted) spectra, but for spectrum assignments, it may lead to the overestimation of the importance of some weak transitions, and controls using, for example, tailored CVS spaces are thus recommended. Finally, we have investigated the basis set requirements of CVS-ADC(2)-x and fc-CVS-EOM-CCSD and conclude that a 6-311++G** basis set provides reasonable relative energies compared to quadruple-ζ reference values. Decontracting the 1s CGTO provides additional accuracy at a small increase in computational cost.

We label this benchmark set as XABOOM and encourage the community to use it for future critical evaluation of methods for calculating X-ray absorption spectra and to critically re-evaluate the conclusions drawn here when more accurate methods become viable. It would be particularly interesting to see how multireference methods perform by comparison. All information necessary for such a comparison—structures, spreadsheets, and SCF energies for control—is available in the Supporting Information.

## Supporting Information Available

Molecular geometries, raw data, additional supporting results; raw data spreadsheet containing total SCF energies, number of MOs, and electrons for each system, results for ADC(2)-x and EOM-CCSD basis set tests, and s → π* transition energies, and oscillator strengths; additional supporting results including tests on using common versus tailored CVS space, scatter plots comparing ADC(2)-x and EOM-CCSD reference data, parameters of utilized exchange–correlation functionals, tabulated results of the full XABOOM benchmark set and of the basis set tests, and relative errors for subsets of the full benchmark set (ZIP)
