# Supplementary material for: XABOOM: An X-ray Absorption Benchmark of Organic Molecules Based on Carbon, Nitrogen, and Oxygen 1s → π* Transitions
Source: J Chem Theory Comput. 2021 Feb 5;17(3):1618–37. doi: 10.1021/acs.jctc.0c01082 (PMC8023667; doi:10.1021/acs.jctc.0c01082)
Supplement: Supplementary file 1 — ct0c01082_si_001.zip [file ct0c01082_si_001.zip › SI.pdf]

Supplementary information for "XABOOM: An X-ray absorption benchmark of organic molecules based on carbon, nitrogen, and oxygen $1s \to \pi^*$ transitions"

Thomas Fransson,* Iulia E. Brumboiu, Marta L. Vidal, Patrick Norman, Sonia Coriani, and Andreas Dreuw*

E-mail: thomas.fransson@fysik.su.se; andreas.dreuw@iwr.uni-heidelberg.de

Molecular structures and raw data are enclosed in separate files. The spreadsheet tabulating transition energies and intensities also include data for the basis set investigation and ground-state SCF energies. Results obtained using a tailored CVS space or equivalent are color-coded in blue.

Systems were selected to ensure a low degree of multireference character, and tests were run to ensure this. SCF LUMO energies and CCSD amplitudes were used as proxies for ground-state multireference character, avoiding systems with (large) negative LUMO energies and/or large T^2 amplitudes. One molecule (1,4-benzoquinone)—which was originally included as system 32—was deemed to have too large degree of multireference character and replaced by 1,2-benzoquinone.

The use of a common versus tailored CVS space or tailored selection of guess vectors is illustrated in Fig. S1. Here the EOM-CCSD results were obtained using default guess vectors and guess vector focusing on transitions from MO 3 or MO 4, where the latter approach thus

selects transitions from specific carbon atoms. For the ADC(2)-x calculations a common CVS space with all four lowest MOs was compared to CVS spaces tailored to MO 3 and MO 4. A small energy shift is noted for EOM-CCSD, as most clearly seen in the difference spectrum. By comparison, for ADC(2)-x the two features which mix in EOM-CCSD are more separate in energy, and no significant mixing occurs—the resulting difference spectrum is close to zero over all shown frequencies. The integrated difference spectrum divided by the integrated full (common CVS or default guess vectors) spectrum for the energy region encompassing the peak max and ±1.5 eV amounts to -0.004 for EOM-CCSD, and 0.003 for ADC(2)-x, illustrating that no significant intensity changes have been introduced in either case. This is despite the major rearrangement of intensity for CCSD when using the default versus manual guess vectors, showing that the use of tailored CVS spaces or guess vectors is stable.

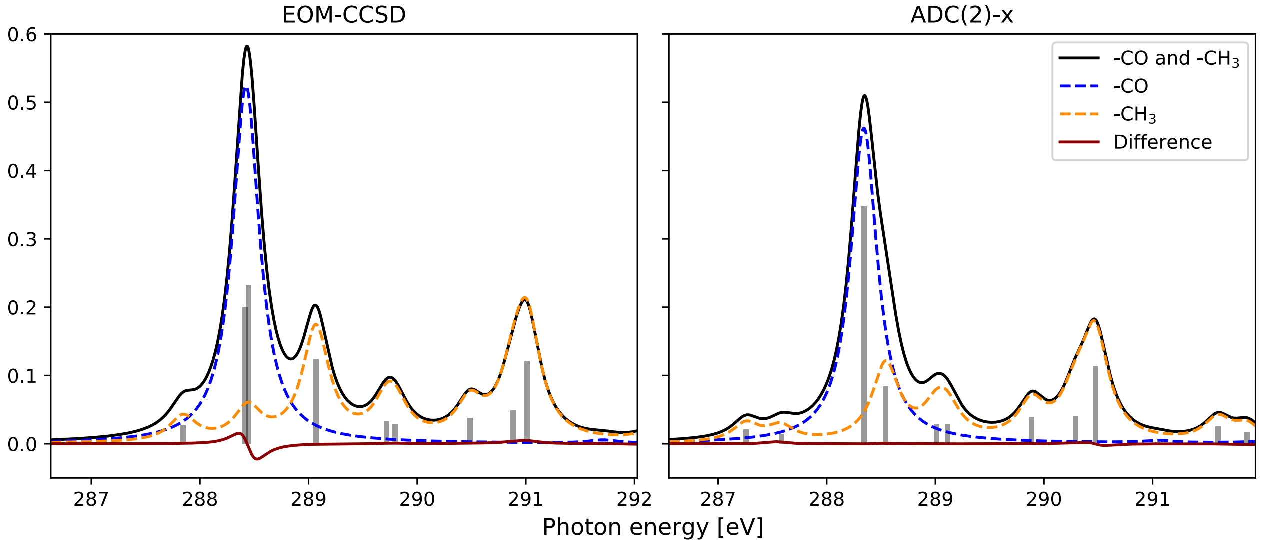

Figure S1: Carbon X-ray absorption spectrum of acetamide (molecule 12), as obtained when considering transitions from both carbon atoms simultaneously, and each atom individually. Including the difference between the summed and total spectra. Partially transparent bars show transition energies and intensities for results obtained with a common CVS space. The spectra have been convoluted with a Lorentzian function of 0.15 eV width.

Fig. S2 shows a scatter-plot of all ADC(2)-x and EOM-CCSD results, showing a generally small spread from the trend lines. Some data points have been labelled, representing large deviations or extremal values.

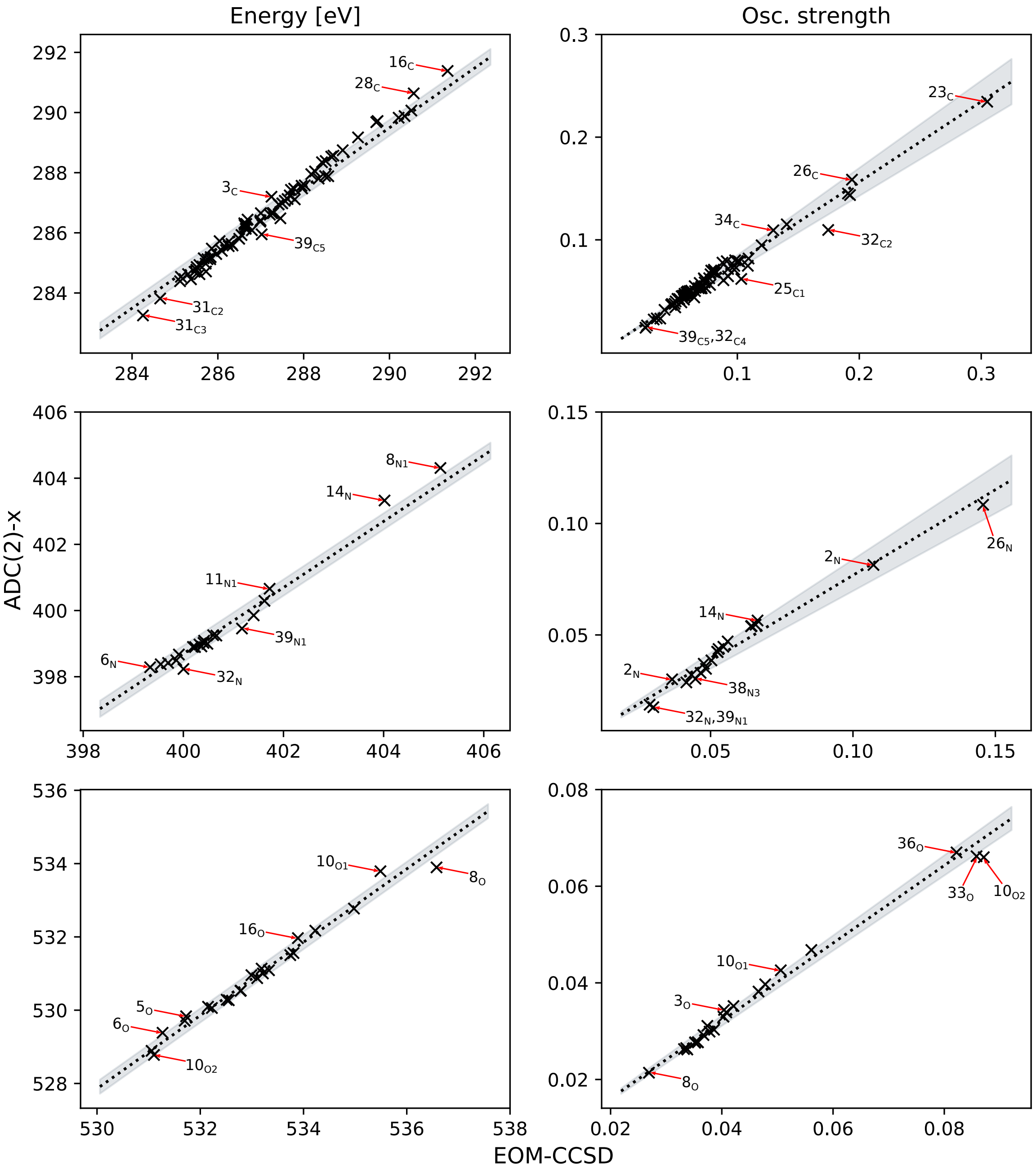

Figure S2: Scatter-plot of ADC(2)-x and EOM-CCSD and $1s \rightarrow \pi^*$ energies and oscillator strengths, as obtained for carbon (top), nitrogen (middle), and oxygen (bottom). Dotted lines indicate mean trend, while the shaded region indicates the standard deviation.

A version of Fig. 3 including only small molecules is illustrated in Fig. S3, comprised of results for systems with up to five heavy (non-hydrogen) atoms—this yields totally 30 states for carbon, 11 for nitrogen, and 14 for oxygen. The resulting weighted error spreads are shown in Table S1, as compared to the values for the full set (Table 1). We see that especially intensity discrepancies are decreased by this reduction of system size, while spreads in energies remain relatively similar.

Table S1: Error spread of excitation energies and intensities, as compared to ADC(2)-x and EOM-CCSD, using the full XABOOM set and a smaller subset. Showing weighted average spreads for all elements in consideration, with energy spread expressed in eV and intensity spread in %.

	Full set				Up to five heavy atoms			
	ADC(2)-x		EOM-CCSD		ADC(2)-x		EOM-CCSD	
	E	I	E	I	E	I	E	I
ADC(1)	1.15	24	0.91	15	1.12	14	0.90	10
ADC(2)	0.29	7	0.35	11	0.27	5	0.32	7
ADC(2)-x	—	—	0.25	8	—	—	0.23	5
ADC(3/2)	0.57	15	0.36	7	0.61	10	0.40	5
CC2	0.25	9	0.25	11	0.25	5	0.25	7
EOM-CCSD	0.25	8	—	—	0.23	6	—	—
PBE	0.56	39	0.66	43	0.47	26	0.52	28
B3LYP	0.30	15	0.30	20	0.28	9	0.25	14
BHandHLYP	0.50	13	0.27	5	0.50	9	0.29	4
$B^{0.58}$LYP	0.60	15	0.36	7	0.59	10	0.38	5
CAM100%	0.29	7	0.17	8	0.30	5	0.18	6
rCAM-B3LYP	0.29	7	0.16	8	0.31	5	0.17	6
CAM-QTP00	0.59	14	0.36	6	0.58	10	0.36	5
SRC2-R1	0.36	11	0.21	8	0.37	7	0.21	6
HCH (PBE)	0.31	21	0.33	26	0.30	16	0.30	19
HCH (BHH)	0.51	20	0.65	26	0.45	17	0.59	21

Fig. S4 illustrates the statistics for the oxygen K-edge, using the full XABOOM set of molecules, as well as a version including DMSO and one excluding three molecules (6, 8, and 10). The inclusion of DMSO yields larger maximum energy deviations for primarily CC2 and ADC(3/2), as well as larger maximum deviations in intensities for most methods. These large discrepancies remain also for basis sets with more diffuse functions (daug-cc-pVTZ), as well as 6-311++G**, but are not present for simpler sulphuroxides such as SO and SO_2. We thus posit that sulphuroxide complexes involving more functional groups

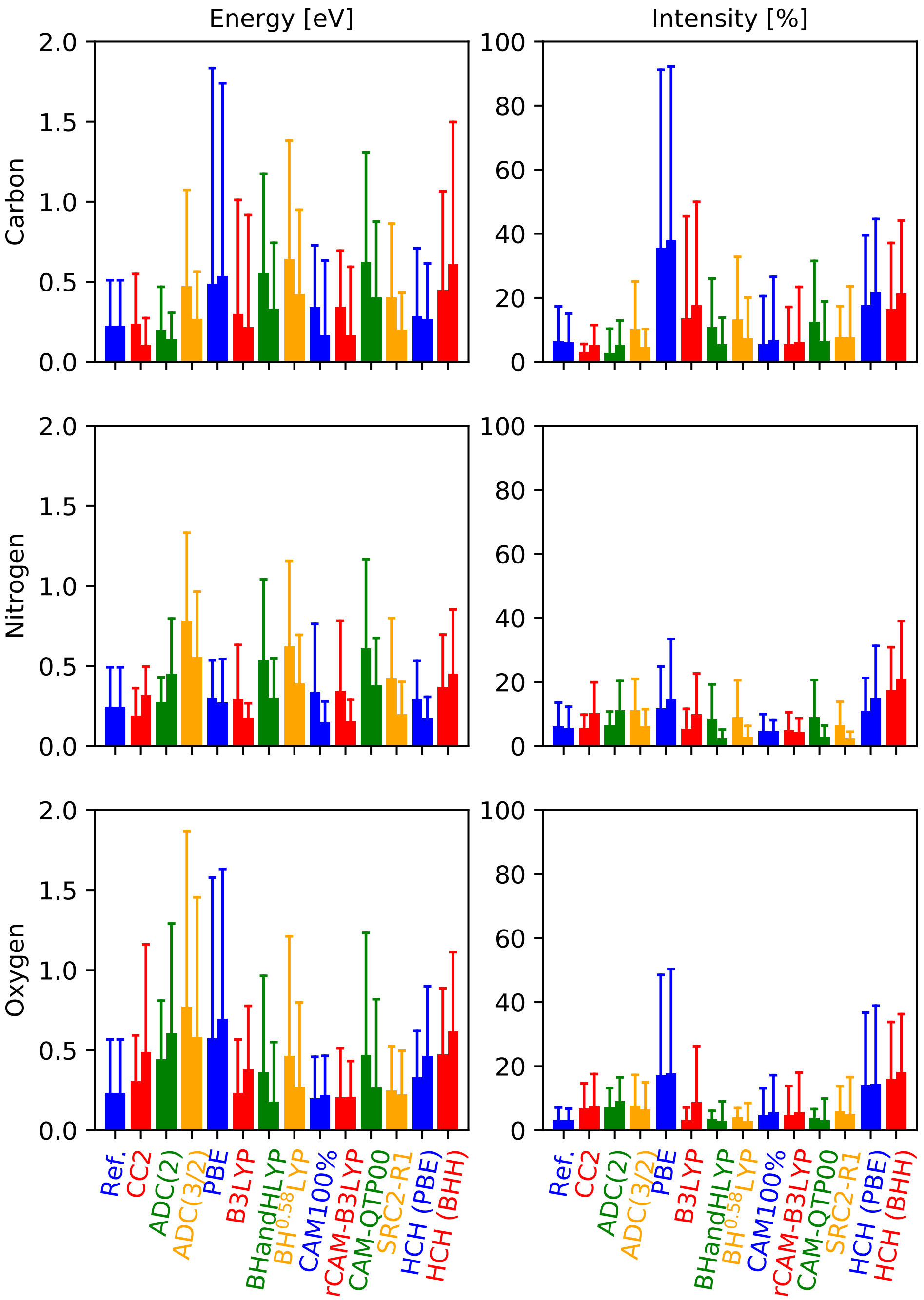

Figure S3: Error spread and maximum absolute deviation considering only systems with up to five heavy (non-hydrogen) atoms. See Fig. 3 for details.

represent a challenge for CC2 and ADC(2) in particular, but not for ADC(2)-x and EOM-CCSD. The bottom panels in Fig. S4 show the statistics for the XABOOM set of molecules excluding nitroxyl, nitrous oxide, and ozone, which leads to significantly lower discrepancies

for all methods, and particularly for CC2, ADC(2), and ADC(3/2). As such, these three molecules represent a family of systems which are extra difficult for both wave function- and DFT-based methods. Furthermore, we note that CC2 exhibits large intensity deviations for cyclohexadione (system 34).

Table S2 contains the parameters of the DFT functionals employed here, for clarity.

Table S2: Parameterization of employed exchange-correlation functionals.

Functional	Ref.	Form	Parameters[a]
PBE	1	$E_x^{PBE} + E_c^{PBE}$	
B3LYP	2	$a_x E_x^{HF} + b_x E_x^{B88} + c_x E_x^{Slater} + a_c E_c^{LYP} + b_c E_c^{VWN}$	$a_x = 0.20$, $b_x = 0.72$, $c_x = 0.08$, $a_c = 0.81$, $b_c = 0.19$
BHandHLYP	3	$a_x E_x^{HF} + b_x E_x^{B88} + E_c^{LYP}$	$a_x = 0.50$, $b_x = 0.50$
$B^{0.58}LYP$	4	$a_x E_x^{HF} + b_x E_x^{B88} + c_x E_x^{Slater} + a_c E_c^{LYP} + b_c E_c^{VWN}$	$a_x = 0.58$, $b_x = 0.39$, $c_x = 0.08$, $a_c = 0.81$, $b_c = 0.19$
CAM100%	5	$a_x^{SR} E_{x,SR}^{HF} + b_x^{SR} E_{x,SR}^{B88} + a_x^{LR} E_{x,LR}^{HF} + b_x^{LR} E_{x,LR}^{B88} + a_c E_c^{LYP} + b_c E_c^{VWN}$	$a_x^{SR} = 0.19$, $b_x^{SR} = 0.81$, $a_x^{LR} = 1.0$, $b_x^{LR} = 0.0$, $a_c = 0.81$, $b_c = 0.19$, $\mu = 0.33$ bohr^{-1}
rCAM-B3LYP	6	$a_x^{SR} E_{x,SR}^{HF} + b_x^{SR} E_{x,SR}^{B88} + c_x E_x^{Slater} + a_x^{LR} E_{x,LR}^{HF} + b_x^{LR} E_{x,LR}^{B88} + a_c E_c^{LYP}$	$a_x^{SR} = 0.1835$, $b_x^{SR} = 0.9498$, $a_x^{LR} = 1.1333$, $b_x^{LR} = 0.0026$, $c_x = -0.1359$, $a_c = 1.0$, $\mu = 0.33$ bohr^{-1}
CAM-QTP00	7	$a_x^{SR} E_{x,SR}^{HF} + b_x^{SR} E_{x,SR}^{B88} + a_x^{LR} E_{x,LR}^{HF} + b_x^{LR} E_{x,LR}^{B88} + a_c E_c^{LYP} + b_c E_c^{VWN}$	$a_x^{SR} = 0.54$, $b_x^{SR} = 0.46$, $a_x^{LR} = 0.91$, $b_x^{LR} = 0.09$, $a_c = 0.80$, $b_c = 0.20$, $\mu = 0.290$ bohr^{-1}
SRC2-R1	4	$a_x^{SR} E_{x,SR}^{HF} + b_x^{SR} E_{x,SR}^{B88} + a_x^{LR} E_{x,LR}^{HF} + b_x^{LR} E_{x,LR}^{B88} + a_c E_c^{LYP} + b_c E_c^{VWN}$	$a_x^{SR} = 0.55$, $b_x^{SR} = 0.45$, $a_x^{LR} = 0.08$, $b_x^{LR} = 0.92$, $a_c = 0.81$, $b_c = 0.19$, $\mu = 0.69$ bohr^{-1}

[a] μ is the range separation parameter that partitions the Coulomb interaction into short- and long-range:[5]

$$\frac{1}{r_{12}} = \frac{1 - [\alpha + \beta \mathrm{erf}(\mu r_{12})]}{r_{12}} + \frac{\alpha + \beta \mathrm{erf}(\mu r_{12})}{r_{12}},$$

where $\alpha = a_x^{SR}$, $1 - \alpha = b_x^{SR}$, $\alpha + \beta = a_x^{LR}$, $1 - \alpha - \beta = b_x^{LR}$.

The statistics for the basis set tests are reported in Table S3 for ADC(2)-x, and in

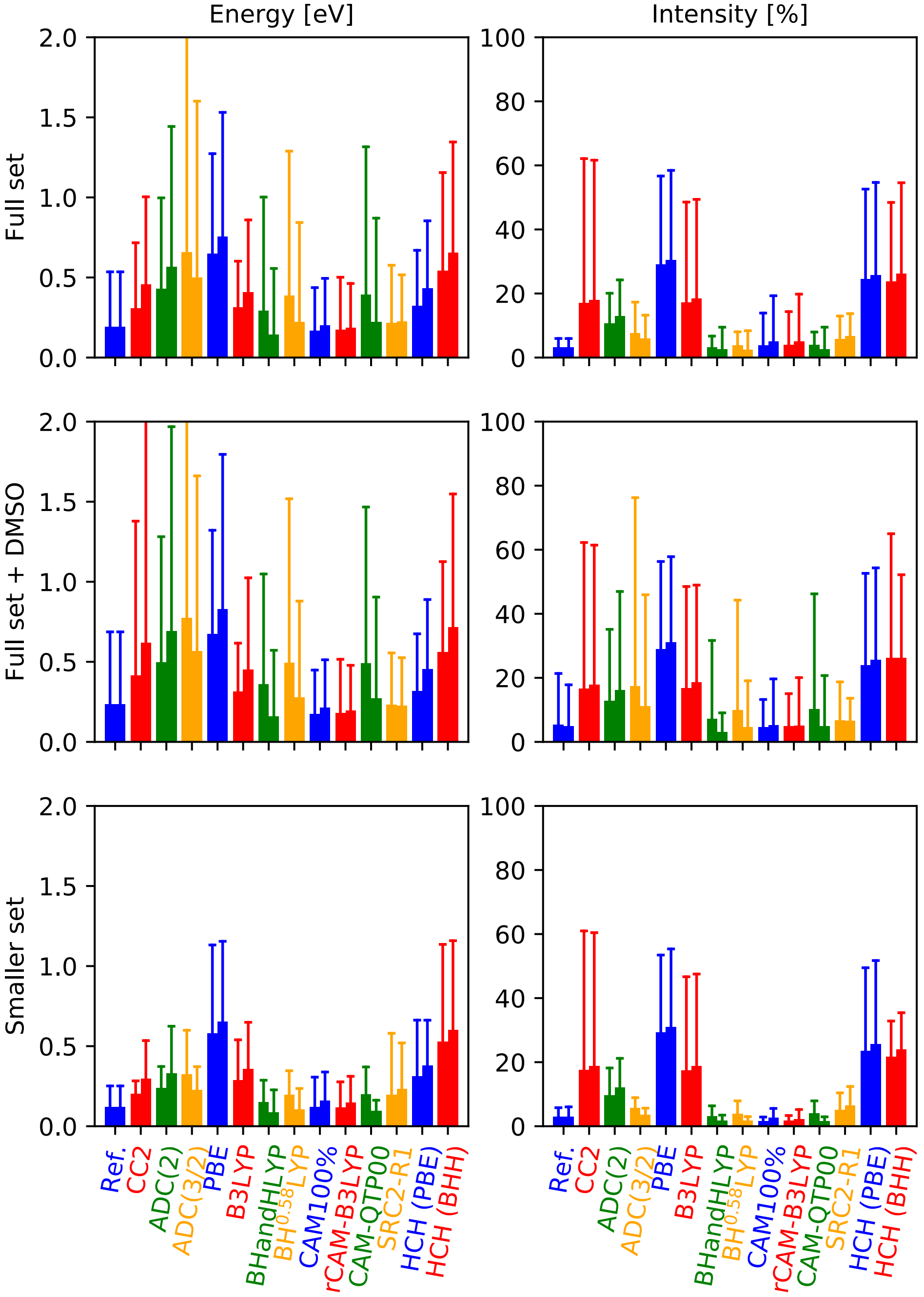

Figure S4: Error spread and maximum absolute deviation for oxygen, including the full benchmark set (top), the full set as well as the DMSO (middle), and the set excluding nitroxyl, nitrous oxide, and ozone (bottom). See Fig. 3 for details.

Table S4 for ADC(2)-x and CCSD using a smaller set of molecules.

The full statistics using ADC(2)-x and EOM-CCSD as the reference are reported in Table S5 and S6, respectively.

Table S3: The basis set dependence of ADC(2)-x, as obtained using aug-cc-pCVQZ/cc-pVQZ results as the reference. Showing mean error (ME), standard deviation (SD), and maximum absolute deviation from the mean (MD), as obtained for the $1s \rightarrow \pi^*$ energies. Also reporting the mean intensity ratio and spread thereof.

		Energy [eV]			Intensity	
Basis set	Element	ME	SD	MD	ME	SD
aCT / T / D	C	0.172	0.014	0.034	0.987	0.005
	N	0.186	0.007	0.015	0.991	0.002
	O	0.214	0.016	0.034	0.993	0.001
aT / T / D	C	0.340	0.013	0.025	0.985	0.007
	N	0.411	0.011	0.021	0.987	0.003
	O	0.510	0.032	0.080	0.989	0.002
u6-311++G**	C	0.526	0.036	0.073	0.979	0.011
	N	0.522	0.030	0.059	0.986	0.005
	O	0.483	0.019	0.033	0.995	0.012
6-311++G**	C	0.725	0.054	0.139	0.986	0.010
	N	0.787	0.024	0.047	0.991	0.005
	O	0.852	0.022	0.038	1.002	0.020

Results obtained from 14 carbon transitions (molecules 3, 4, 5, 7, 15, 16, 19, 20, and 27), 10 nitrogen transitions (molecules 6, 7, 8, 11, 14, 19, 20, and 27), and 10 oxygen transitions (molecules 3, 5, 6, 10, 14, 16, and 23).

Table S4: The basis set dependence of ADC(2)-x and EOM-CCSD, as obtained using aug-cc-pCVQZ/cc-pVQZ results as the reference. See Table S3 for details.

		ADC(2)-x			EOM-CCSD		
Basis set	Element	ME	SD	MD	ME	SD	MD
aCT / T / D	C	0.174	0.018	0.032	0.209	0.014	0.024
	N	0.182	0.002	0.003	0.218	0.004	0.005
	O	0.210	0.013	0.024	0.255	0.012	0.018
aT / T / D	C	0.342	0.014	0.022	0.519	0.010	0.017
	N	0.406	0.007	0.013	0.608	0.028	0.048
	O	0.500	0.018	0.034	0.725	0.030	0.054
u6-311++G**	C	0.525	0.049	0.075	0.579	0.037	0.062
	N	0.512	0.028	0.043	0.572	0.023	0.039
	O	0.478	0.018	0.024	0.531	0.040	0.070
6-311++G**	C	0.732	0.075	0.132	0.953	0.072	0.120
	N	0.779	0.022	0.027	1.056	0.022	0.037
	O	0.843	0.017	0.026	1.152	0.046	0.084

Results obtained from 7 carbon transitions (molecules 3, 4, 5, 7, and 15), 6 nitrogen transitions (molecules 6, 7, 8, and 11), and 7 oxygen transitions (molecules 3, 5, 6, 10, and 14).

Table S5: Mean error (ME), standard deviation (SD), and maximum absolute deviation from the mean (MD), using ADC(2)-x as the reference. See Eqs. 9–14 for definition of statistics.

		carbon		nitrogen		oxygen	
		E	I	E	I	E	I
CCSD	ME	0.51	1.29	1.31	1.31	2.14	1.25
	SD	0.27	10	0.25	10	0.19	3
	MD	0.58	33	0.61	29	0.54	6
ADC(1)	ME	9.50	2.56	13.14	2.53	17.02	2.22
	SD	1.19	30	1.16	24	1.00	7
	MD	2.48	142	2.59	73	3.17	13
ADC(2)	ME	3.36	1.28	3.42	1.08	2.76	0.94
	SD	0.22	5	0.35	8	0.43	11
	MD	0.43	27	0.65	21	1.00	20
ADC(3/2)	ME	1.87	1.33	3.51	1.48	5.64	1.46
	SD	0.50	15	0.73	19	0.66	8
	MD	1.19	55	1.63	57	2.05	17
CC2	ME	2.28	1.14	2.43	1.01	2.10	0.88
	SD	0.25	8	0.21	6	0.31	17
	MD	0.54	36	0.48	14	0.72	62
PBE	ME	-17.11	0.45	-19.03	0.62	-21.90	0.68
	SD	0.51	43	0.64	35	0.65	29
	MD	1.52	89	1.04	77	1.27	57
B3LYP	ME	-10.75	0.84	-11.79	1.00	-13.46	0.95
	SD	0.31	17	0.28	7	0.31	17
	MD	0.88	48	0.52	16	0.60	49
BHandHLYP	ME	-2.47	1.53	-1.81	1.62	-1.45	1.44
	SD	0.57	16	0.48	15	0.29	3
	MD	1.22	61	1.23	51	1.00	7
$B^{0.58}LYP$	ME	1.28	1.72	2.60	1.77	3.72	1.55
	SD	0.68	18	0.58	17	0.39	4
	MD	1.44	56	1.42	57	1.29	8
CAM100%	ME	-10.69	1.04	-11.70	1.13	-13.23	1.06
	SD	0.33	8	0.26	5	0.17	4
	MD	0.73	28	0.81	12	0.44	14
rCAM-B3LYP	ME	-10.42	1.06	-11.37	1.13	-12.84	1.06
	SD	0.34	8	0.27	5	0.17	4
	MD	0.72	26	0.85	13	0.50	14
CAM-QTP00	ME	-1.46	1.65	-0.52	1.71	0.17	1.51
	SD	0.66	17	0.58	16	0.39	4
	MD	1.39	56	1.43	52	1.32	8
SRC2-R1	ME	0.26	1.36	0.72	1.53	0.91	1.35
	SD	0.41	11	0.33	13	0.22	6
	MD	0.85	44	0.82	50	0.58	13
HCH (PBE)	ME	-0.24	0.41	0.36	0.44	0.60	0.38
	SD	0.32	21	0.27	16	0.32	25
	MD	0.79	52	0.54	25	0.67	53
HCH (BHH)	ME	3.43	0.40	3.96	0.42	4.00	0.34
	SD	0.48	18	0.60	24	0.54	24
	MD	1.38	41	1.04	50	1.16	48

Table S6: Mean error (ME), standard deviation (SD), and maximum absolute deviation from the mean (MD), using EOM-CCSD as the reference. See Eqs. 9–14 for definition of statistics.

		carbon		nitrogen		oxygen	
		E	I	E	I	E	I
ADC(2)-x	ME	-0.51	0.78	-1.31	0.77	-2.14	0.80
	SD	0.27	9	0.25	9	0.19	3
	MD	0.58	25	0.61	23	0.54	6
ADC(1)	ME	8.99	1.96	11.83	1.90	14.87	1.78
	SD	0.93	19	0.92	13	0.82	5
	MD	1.98	84	1.97	37	2.72	10
ADC(2)	ME	2.85	1.00	2.11	0.84	0.62	0.75
	SD	0.22	9	0.55	16	0.57	13
	MD	0.62	41	1.01	40	1.44	24
ADC(3/2)	ME	1.36	1.03	2.20	1.12	3.49	1.17
	SD	0.27	6	0.50	9	0.50	6
	MD	0.66	27	1.02	22	1.60	13
CC2	ME	1.77	0.89	1.12	0.78	-0.04	0.71
	SD	0.15	8	0.38	14	0.46	18
	MD	0.37	39	0.75	34	1.00	62
PBE	ME	-17.62	0.36	-20.33	0.49	-24.05	0.55
	SD	0.59	47	0.77	40	0.75	30
	MD	1.40	90	1.39	79	1.53	58
B3LYP	ME	-11.26	0.66	-13.10	0.77	-15.60	0.77
	SD	0.25	22	0.34	15	0.41	18
	MD	0.76	55	0.72	33	0.86	49
BHandHLYP	ME	-2.97	1.18	-3.12	1.23	-3.60	1.15
	SD	0.31	7	0.24	5	0.14	3
	MD	0.75	23	0.61	17	0.56	9
$B^{0.58}LYP$	ME	0.77	1.33	1.29	1.34	1.57	1.24
	SD	0.41	9	0.34	7	0.22	2
	MD	0.98	28	0.80	22	0.84	8
CAM100%	ME	-11.20	0.81	-13.00	0.86	-15.38	0.85
	SD	0.15	9	0.18	7	0.20	5
	MD	0.61	43	0.38	21	0.49	19
rCAM-B3LYP	ME	-10.92	0.82	-12.68	0.87	-14.98	0.85
	SD	0.15	9	0.17	7	0.19	5
	MD	0.59	42	0.37	20	0.46	20
CAM-QTP00	ME	-1.97	1.28	-1.83	1.29	-1.98	1.21
	SD	0.40	8	0.34	6	0.22	3
	MD	0.92	28	0.82	18	0.87	9
SRC2-R1	ME	-0.25	1.06	-0.59	1.16	-1.23	1.09
	SD	0.20	9	0.20	5	0.23	7
	MD	0.48	32	0.50	16	0.52	14
HCH (PBE)	ME	-0.75	0.32	-0.94	0.34	-1.55	0.30
	SD	0.29	26	0.33	23	0.43	26
	MD	0.81	56	0.72	32	0.85	55
HCH (BHH)	ME	2.92	0.32	2.65	0.33	1.86	0.27
	SD	0.63	24	0.75	31	0.65	26
	MD	1.84	51	1.30	59	1.35	55

References

(1) Perdew, J. P.; Burke, K.; Ernzerhof, M. Generalized Gradient Approximation Made Simple. *Phys. Rev. Lett.* **1996**, *77*, 3865–3868.

(2) Becke, A. D. Density-Functional Thermochemistry. III. The Role of Exact Exchange. *J. Chem. Phys.* **1993**, *98*, 5648–5652.

(3) Becke, A. D. A new mixing of Hartree-Fock and local density-functional theories. *J. Chem. Phys.* **1993**, *98*, 1372–1377.

(4) Besley, N. A.; Peach, M. J. G.; Tozer, D. J. Time-dependent density functional theory calculations of near-edge X-ray absorption fine structure with short-range corrected functionals. *Phys. Chem. Chem. Phys.* **2009**, *11*, 10350–10358.

(5) Yanai, T.; Tew, D. P.; Handy, N. C. A new hybrid exchange-correlation functional using the Coulomb-attenuating method (CAM-B3LYP). *Chem. Phys. Lett.* **2004**, *393*, 51–57.

(6) Cohen, A. J.; Mori-Sánchez, P.; Yang, W. Development of exchange-correlation functionals with minimal many-electron self-interaction error. *J. Chem. Phys.* **2007**, *126*, 191109.

(7) Verma, P.; Bartlett, R. J. Increasing the applicability of density functional theory. IV. Consequences of ionization-potential improved exchange-correlation potentials. *J. Chem. Phys.* **2014**, *140*, 18A534.
